# D–A Structural Oligomers Containing Benzothiadiazole or Benzophenone as Novel Multifunctional Materials for Electrochromic and Photodetector Devices

**DOI:** 10.3390/polym15102274

**Published:** 2023-05-11

**Authors:** Zipeng He, Binhua Mei, Hongmei Chu, Yanjun Hou, Haijun Niu

**Affiliations:** 1 Key Laboratory of Chemical Engineering Process and Technology for High-Efficiency Conversion, College of Heilongjiang Province, Heilongjiang University, Harbin 150080, China; 2 Key Laboratory of Functional Inorganic Material Chemistry, Ministry of Education of the People’s Republic of China, Heilongjiang University, Harbin 150080, China

**Keywords:** electrochromic, photodetector, D–A structure

## Abstract

In this study, six conjugated oligomers containing D–A structures were synthesized using the Stille coupling reaction and named PHZ1–PHZ6. All the oligomers utilized demonstrated excellent solubilities in common solvents and notable color variations in the domain of electrochromic characteristics. By designing and synthesizing two electron-donating groups modified with alkyl side chains and a common aromatic electron-donating group, as well as cross-binding them with two electron-withdrawing groups with lower molecular weights, the six oligomers presented good color-rendering efficiencies, among which PHZ4 presented the best color-rendering efficiency (283 cm^2^·C^−1^). The products also demonstrated excellent electrochemical switching-response times. PHZ5 presented the fastest coloring time (0.7 s), PHZ3 and PHZ6 presented the fastest bleaching times (2.1 s). Following 400 s of cycling activity, all the oligomers under study showed good working stabilities. Moreover, three kinds of photodetectors based on conducting oligomers were prepared, and the experimental results show that the three photodetectors have better specific detection performances and gains. These characteristics indicate that oligomers containing D–A structures are suitable for use as electrochromic and photodetector materials in the research.

## 1. Introduction

In the new phase of science and technology, there is a high demand for relevant materials. The design of materials in the research is not limited to the requirement of their single, stable performance, but aims to make them meet a variety of requirements, such as the development of environmental protection procedures, simple synthesis, and excellent versatility [1,2,3]. Organic color-changing materials are environmentally friendly and consume less energy. They are a type of natural material and are useful for practical applications [4,5,6]. Organic photodetector materials offer a very important application prospect for the connection between light and electricity [7,8,9,10]. Using natural renewable resources to create a range of industrial products can considerably reduce energy consumption levels. Organic conductive oligomer materials have various properties, including their stability and mature synthesis technology, and play a unique role in the fields of optics and electricity [11,12,13].

The most basic principle of electrochromic materials is their electron-transfer activity, in which molecules gain and lose electrons through REDOX reactions, presenting reversible color changes at different voltage values [14,15,16,17]. In the research, the process of natural selection is an inevitable process when developing materials; from the beginning stage of simple inorganic discoloration to the present organic oligomer’s structure, the field of electrochromic materials has experienced dramatic changes [18,19,20]. Organic conductive oligomer materials are very important photoelectric components. Their stable conjugated system and excellent photoelectric properties provide them with a unique status in the field of organic synthesis. At present, electrochromic materials can be perceived everywhere; whether this be in a professional environment, such as an office space where computer programs include Windows [21], a smartphone, which can be used by everyone [22], or mechanical precision LED devices [23], increasingly more electrochromic devices using novel structures and comprehensive functions are appearing, and have become an indispensable part of peoples’ daily lives.

From the many conductive oligomer materials available, the conjugated structure that is produced following the combination of electron-absorbing and electron-giving groups produced better photoelectric properties; therefore, the six oligomers used in this study present the characteristic structure that is commonly known in the research as the D–A structure [24]. At the same time, we tested the related properties of the photodetectors for three oligomers under study. The results show that the materials have certain application prospects in the field. It is obvious that large molecules with high-quality conducting molecular oligomers are also luminescent and become a heat source worth investigating in the field of photodetectors.

## 2. Experimental

### 2.1. Materials

N-methyl pyrrolidone, potassium carbonate (anhydrous), bromo-isooctane, tetrabutylammonium perchlorate, phenylenediamine, thionyl chloride, ethyl acetate, dimethyl sulfoxide, 1,4-dibromonaphthalene, tetrahydrofuran, petroleum ether, toluene, dichloromethane, acetonitrile N, n-dimethylformamide, anhydrous magnesium sulfate, n-bromo-succinimide (NBS), tetraphenyl phosphine palladium, potassium iodide, anhydrous calcium chloride, potassium hydroxide, fluorene, ferric chloride, tributyl tin chloride, butyl lithium, 18-crown ether-6, and other chemicals involved in this study were purchased from Aladdin Reagent Co. Ltd. and used directly for the synthesis process without being exposed to any purification stages.

### 2.2. Characterization

Bruker’s AC-400 MHz spectrometer (Bruker, Billerica, MA, USA) was used to obtain the NMR hydrogen spectra of the oligomers in the experiment. Fourier transform infrared spectroscopies (FT-IRs) were measured using a PerkinElmer Spectrum Two spectrometer (PerkinElmer, Inc., Waltham, MA, USA) (400–4000 cm^−1^). A Shimadzu UV-2501 spectrophotometer (Shimadzu Europa GmbH, Duisburg, Germany) was used to obtain the UV–vis spectra of the oligomers. A CHI 660 electrochemical workstation (Shanghai Chenhua Co., Ltd., Shanghai, China) was used to perform cyclic voltammetry testing in a 0.2 mol·L^−1^ CH_3_CN solution of TBAP with a scan rate of 50 mV·s^−1^. A conventional three-electrode system was used to obtain the electrochromic properties of the oligomers. The reference and counter electrodes were saturated with potassium chloride (KCl)-Ag/AgCl and a Pt electrode, respectively. The surface morphologies of the oligomers were observed using an SHL3-NanoFirst-2000-AFM. The density functional theory was assessed by using SGI Origin 350 server using Gaussian 03 software (Gaussian, Inc, New Hampshire, USA) and the B3LYP method. The spectro-electrochemistry and kinetic properties of the oligomers were tested by coupling a UV–vis absorption spectrometer with an electrochemical workstation. The molecular weights of the oligomers under study were measured by gel permeation chromatography (GPC) with a Waters 2690 instrument. A plasma cleaner was used to clean the ITO glasses. The measurement of EQE was performed on a system obtained from Beijing 7-Star Optical Instruments Co. The dark current density–voltage data were obtained from a Keithley 236 Source Measure unit.

### 2.3. Preparation of Oligomer Films and the Fabrication and Characterization of the Photodetector Devices

Preparation process of oligomer films: ITO conductive glasses needed to be treated prior to creating the films. Acetone, ethanol, and deionized water were used to ultrasonically clean the ITO conductive glasses.

Device fabrication: A mixture containing the oligomer and PC_61_BM, with a weight ratio of 1:2, was mixed into a solution of chlorobenzene at a concentration of 10 mg·mL^−1^, heated to 60 °C, and then stirred for 3 h as an example of a photodetector based on the oligomer. The active layer produced was approximately 120 nm thick.

Device characterization: To avoid the occurrence of electrostatic interference in the air, during all measurements performed under ambient circumstances, the unencapsulated devices were housed in a metal enclosure (see Supplementary Materials).

### 2.4. Synthesis of the Compounds

In this study, four monomers and six conductive oligomers were synthesized, and the compounds were characterized by a series of tests.

**Scheme 1:**
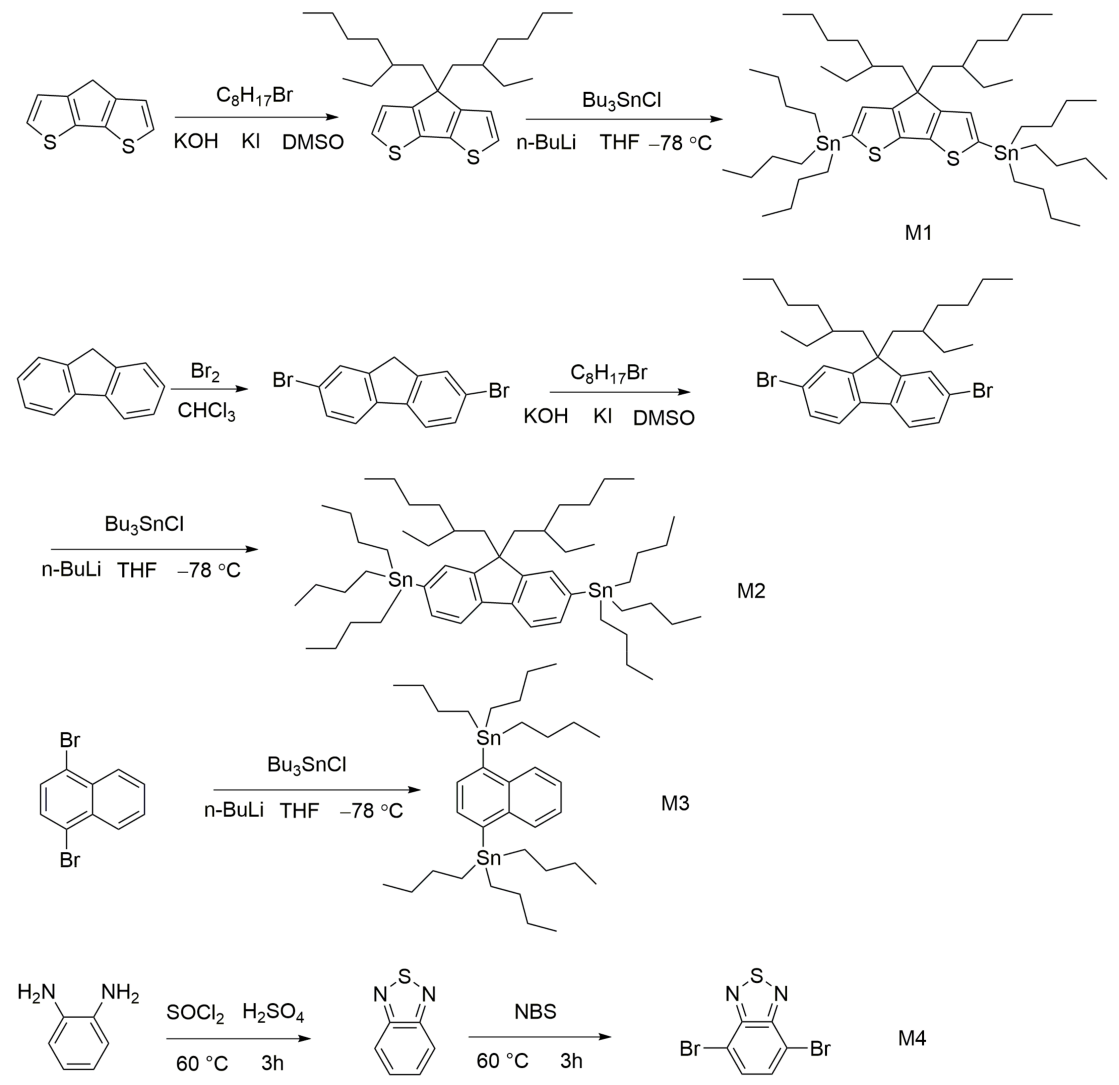
Synthesis routes of M1–M4.

Scheme 1 describes the synthesis process of the four monomers in detail.

#### 2.4.1. Synthesis of M1

In the experiment, we used a 250 mL single-neck round-bottomed flask, added cyclopententhiophene (1.78 g, 10 mmol) and DMSO (50 mL) into it, stirred the solution well, and then added potassium iodide (0.05 g, 8 mmol) and potassium hydroxide (2 g, 36 mmol). Then, we added bromo-isooctane (0.05 g, 8 mmol) in a drop-by-drop manner, and finally added a small quantity of 18-crown ether-6, reacted at room temperature for 24 h, poured it into the water, extracted petroleum ether from the solution several times, added anhydrous calcium chloride to dry the solution, filtered it, and then spun steam to obtain a crude product. The crude product was then purified by petroleum ether silica gel column chromatography to obtain a pure yellowish oily liquid (4.7 g (84%)). ^1^H NMR (400 MHz, chloroform-d) δ 7.08 (d, J = 4.9 Hz, 2H), 6.92 (dt, J = 4.8, 2.3 Hz, 2H), 1.9–1.8 (m, 4H), 0.96 (ddt, J = 32.9, 11.9, 6.0 Hz, 18H), 0.74 (t, J = 6.8 Hz, 6H), 0.6 (d, J = 7.2 Hz, 6H).

The yellowish oily liquid (2.8 g, 5 mmol) and 30 mL of anhydrous THF were added into a 250 mL three-necked round-bottomed bottle, cooled to −78 °C, and kept at this temperature. Then, butyl lithium (8 mL, 20 mmol, 2.5 mol·L^−1^) was added to the bottle in a drop-by-drop manner. Following 30 min of the reaction process, tributyltin chloride (6.8 mL, 25 mmol) was slowly dripped into the remaining solution. Following this process, the cooling device was removed, and the reaction temperature was slowly increased to room temperature for an additional 2 h. All the abovementioned experimental operations were conducted in a nitrogen atmosphere. Following the reaction, the product was washed in deionized water, several times, to separate the organic phase from the solution. Then, tetrahydrofuran was spun out under pressure to obtain 4.4 g (90%) of brown-red oily liquid without being subjected to further purification processes.

#### 2.4.2. Synthesis of M2

We added fluorene (5.0 g, 30 mmol) and 50 mL of CHCl_3_ to a 100 mL single-neck round-bottomed flask containing a stirrer wrapped in aluminum foil. The solution was cooled to 0 °C, and then ferric chloride (0.0716 g, 0.45 mmol) was added to it, followed by the gradual addition of liquid bromine (3.26 mL, 63.2 mmol). The drip time should be controlled for a period of at least 30 min to allow the substance to fully react. After the operation, the solution should be heated slowly until it is restored to room temperature. The mixed solution was stirred and reacted for an entire day under these conditions, washed with a Na_2_S_2_O_3_ saturated solution several times, and then repeatedly extracted with CHCl_3_ until the solution was colorless. Finally, the organic solvent was spun out to obtain 9.4 g (97%) of a white solid solution.

According to the 4,4-bis(2-ethylhexyl)-4h-cyclopentadithiophene experimental process, the dark oily liquid was synthesized using 50 mL of DMSO, 2, 7-dibromo-9h-fluorene (3.24 g, 10 mmol), bromo-isooctane (4.42 g, 23 mmol), potassium iodide (0.05 g, 8 mmol), respectively, potassium hydroxide (2 g, 36 mmol), and 18-crown ether-6. The yield was 4.4 g (80%).

According to the method of M1, M2 was synthesized using the following materials: 30 mL of THF, 2,7-dibromo-9,9-bis (2-ethylhexyl)-9h-fluorene (2.74 g, 5 mmol), butyllithium (8 mL, 20 mmol, 2.5 mol·L^−1^), and tributyl tin chloride (6.8 mL, 25 mmol). The M2 product was a dark-brown liquid weighing 4.2 g (87%). ^1^H NMR (400 MHz, chloroform-d) δ 7.6–7.5 (m, 2H), 7.15 (d, J = 1.6 Hz, 2H), 7.13–7.05 (m, 2H), 2.65 (t, J = 7.5 Hz, 4H), 1.95 (dt, J = 11.8, 5.7 Hz, 4H), 1.64 (q, J = 7.7 Hz, 12H), 1.37–1.31 (m, 18H), 0.95–0.70 (m, 42H), 0.53–0.48 (m, 8H).

#### 2.4.3. Synthesis of M3

According to the reaction process of the first two monomers, the preparation method of M3 was the same as that mentioned above: 30 mL of THF, 1,4-dibromonaphthalene (1.43 g, 5 mmol), butyllithium (8 mL, 20 mmol, 2.5 mol·L^−1^), and tributyl tin chloride (6.8 mL, 25 mmol). The product was a white oily liquid weighing 2.68 g (62%).

#### 2.4.4. Synthesis of M4

SOCl_2_ (100 mL) was added to a 250 mL three-neck round-bottomed flask and o-phenylenediamine (10.8 g, 0.1 mol) was evenly dissolved into the solvent. After stirring for 10 min, we slowly added 1 mL of concentrated H_2_SO_4_ into the flask using an eyedropper. Equipped with a tail gas treatment device, the temperature increased to 60 °C for 3 h. After being exposed to a natural-cooling process, the reaction liquid was poured into a large quantity of icy water, which was then filtered and recrystallized to obtain a white solid solution weighing 11.83 g (87%). The white solid product (6.8 g, 0.05 mol) was uniformly dissolved in hydrobromic acid (40%, 90 mL) and stirred continuously; then, it was gradually added to a Br_2_ solution (24 g, 1.5 mol) of hydrobromic acid (40%, 100 mL) in a ventilated environment. After 6 h, yellow precipitates were attached to the bottle. After being naturally cooled to reach room temperature, it was washed with a saturated NaHSO_3_ solution several times [25]. After the solution was clarified, it was filtered and washed in deionized water several times and dried using anhydrous Na_2_SO_4_. The final product was a gray solid weighing 13.2 g (90%). ^1^H NMR (400 MHz, chloroform-*d*) δ 7.74 (s, 2H).

### 2.5. Synthesis of the Oligomers

**Scheme 2:**
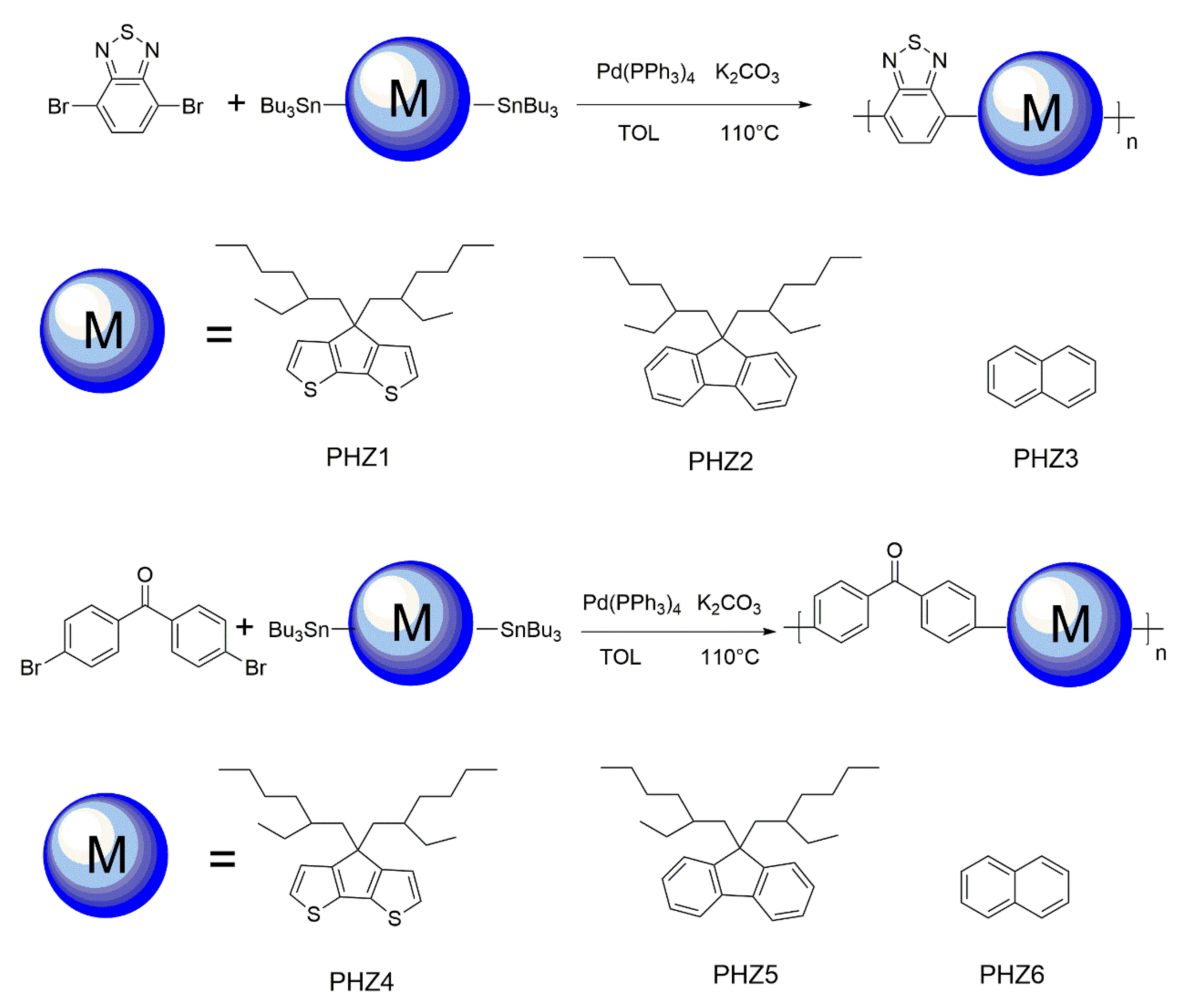
Synthesis methods for six oligomers.

Scheme 2 presents six detailed synthesis methods for the oligomers, all of which were prepared using the Stille coupling reaction. We used PHZ1 as an example to list the specific synthesis steps.

Monomer M1 (2.45 g, 2.5 mmol), 4,7-dibromo-2,1,3-benzothiadiazole (M4) (0.59 g, 2 mmol), an anhydrous K_2_CO_3_ solution (3 mL, 3 mol·L^−1^), and 15 mL of toluene were added to a 100 mL three-neck round-bottomed flask for ultrasonic degassing for 30 min. Finally, Pd (PPh_3_)_4_ (6.96 mg, 0.006 mmol) was also added to it. Reflux heating in a nitrogen atmosphere for 3 days was performed. Following the reaction process, the reaction liquid was repeatedly washed with deionized water, and the organic phase was obtained by separating the liquid. The organic phase was added to 100 mL of an icy methanol solution in a drop-by-drop manner under the condition of agitation, and the crude product produced was filtered. We used a Soxhlet extractor to extract the methanol for 5 days and dried the solution for 12 h under vacuum at 80 °C. PHZ1 was an orange solid; yield: 0.87 g (63%).

PHZ2 was a purple solid (0.69 g, 61%), PHZ3 was a gray solid (0.56 g, 52%), PHZ4 was a yellow solid (0.76 g, 62%), PHZ5 was a light-yellow solid (0.73 g, 63%), and PHZ6 was a white solid (0.67 g, 58%).

## 3. Results and Discussion

### 3.1. Infrared Spectrogram Analysis

**Figure 1:**
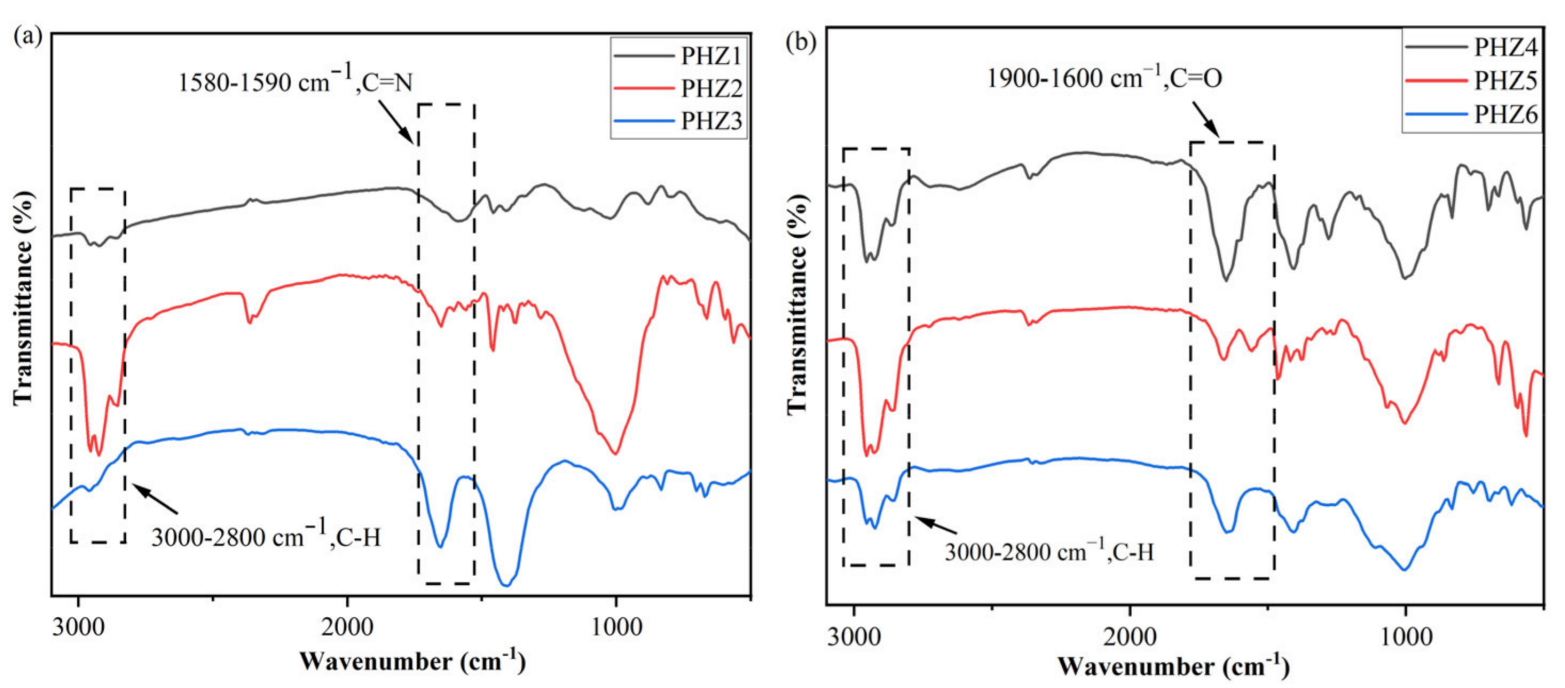
The FT-IR spectra of PHZ1–PHZ3 (**a**) and the FT-IR spectra of PHZ4–PHZ6 (**b**).

By performing infrared analyses of the six newly synthesized oligomers, the chemical bonds and vibration types were successfully identified, and the structures of the new oligomers were further characterized. As shown in Figure 1, the infrared spectra of the six oligomers were divided into two groups for analysis from the perspective of structural similarity. The first three oligomers showed obvious absorption peaks of approximately 3000 cm^−1^, whereas characteristic peaks of approximately 1000 cm^−1^ were also evident, which proved that the oligomers possessed a benzene-ring structure [26]. The value of 1580–1590 cm^−1^ is the standard position of the C=N bond. Specific absorption peaks can be observed in the figure; therefore, we proved that the oligomer possessed a benzothiadiazole structure. In summary, the oligomer structures are consistent with the experimental designs. The characteristic functional groups of the last three oligomers presented benzene-ring and carbon–oxygen double-bond structures, which showed obvious peaks at 3000–2800 and 1900–1600 cm^−1^, respectively, in the infrared spectrum; therefore, the oligomers were confirmed to be successfully synthesized.

### 3.2. Solubility Values and Gel-Permeation Chromatographies of Oligomers

We dissolved 10 mg of the oligomer in 1 mL of organic solvent and observed whether the solution was uniform and stable to judge its solubility [27]. According to the experimental results, the six oligomers can form stable solutions in common organic solvents with good solubility properties (Table 1). This outcome may have been due to the low molecular weight of PHZ1–PHZ6, which is easily dispersed in the solvent. Gel-permeation chromatography showed the degree of polymerization of the oligomers (Table 2), and the results show that the degrees of oligomerization of the six oligomers are not very different, possibly due to the same reaction temperature and reaction time relationships they exhibited.

### 3.3. Surface Morphologies of Oligomers

**Figure 2:**
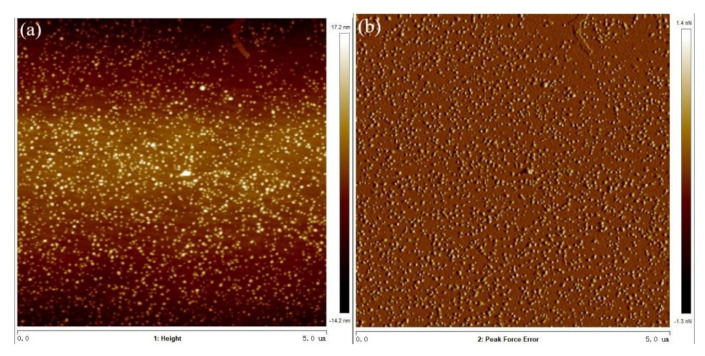

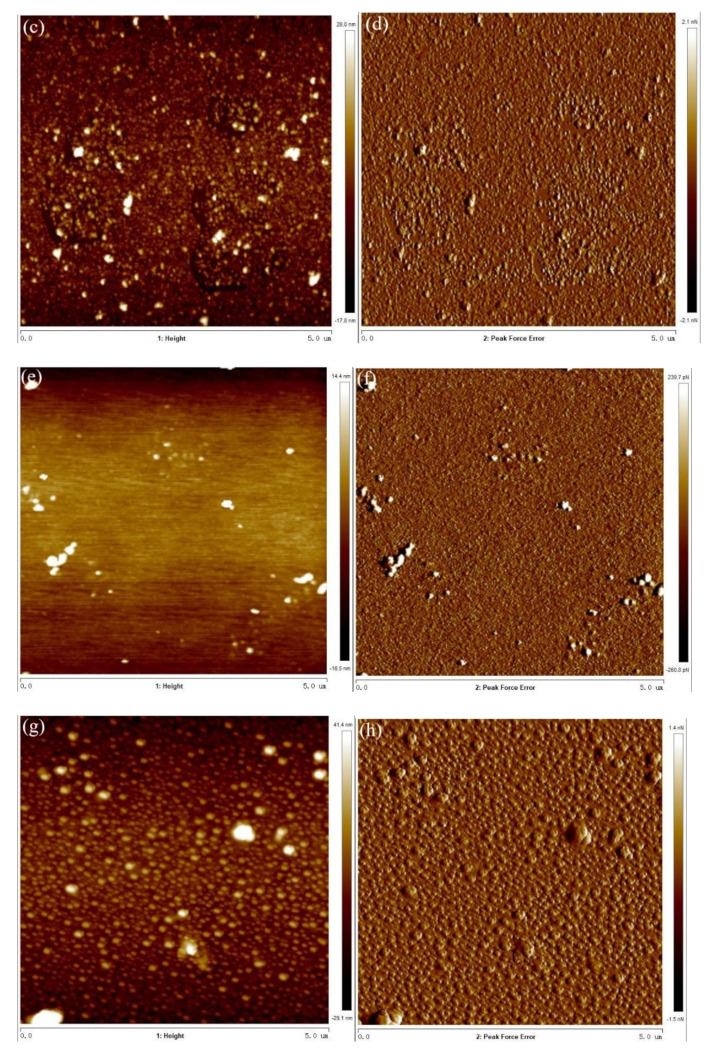

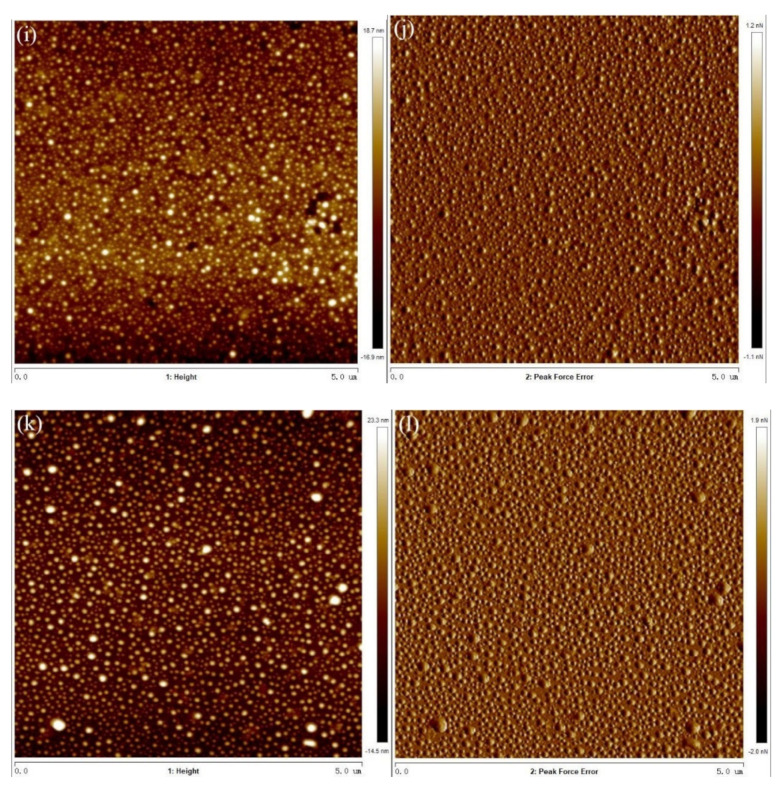
AFM images of oligomer films: (**a**,**b**) PHZ1; (**c**,**d**) PHZ2; (**e**,**f**) PHZ3; (**g**,**h**) PHZ4; (**i**,**j**) PHZ5; (**k**,**l**) PHZ6.

A high-precision scanning probe microscope called atomic force microscopy (AFM) was used to examine the surface morphologies of the nanometer-sized oligomer films. The surface micromorphologies of the oligomer films are shown in Figure 2. The surface roughness values of PHZ1–PHZ6 were 4.54, 5.71, 4.09, 9.74, 4.44, and 5.65 nm, respectively, and the aggregation state of each oligomer was different. PHZ4 had the roughest surface, whereas PHZ5 and PHZ6 had homogeneity values similar to the previous three oligomers, indicating that the reactive molecules of benzophenone and cyclopententhiophene had a high aggregation level in addition to the influence of the solution’s concentration. Rougher surface micromorphologies can speed up the electron migration process and shorten electrochromic response times [28].

### 3.4. Optical Properties

**Figure 3:**
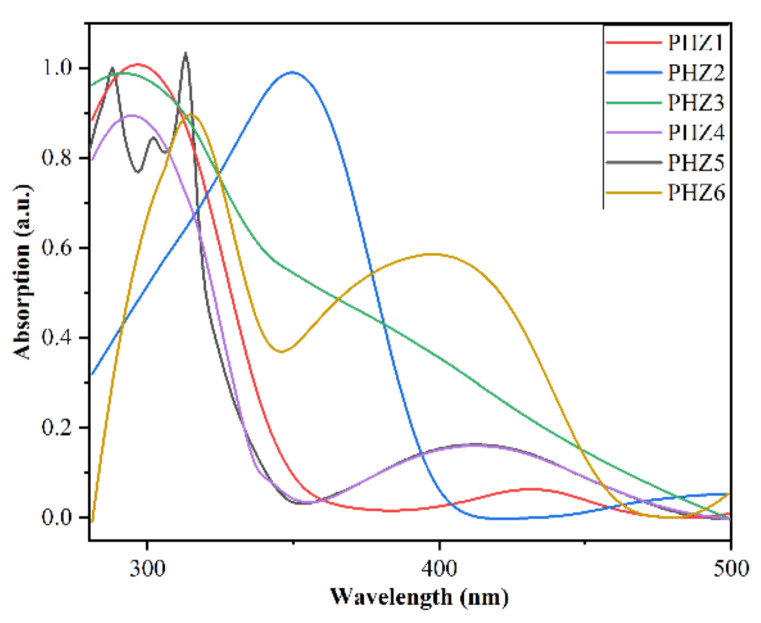
UV–vis spectra of six oligomers in DMF.

Figure 3 presents the detailed UV-absorption values of the oligomers exposed to 280–500 nm. Each oligomer presented different maximum absorption wavelengths (Table 3). PHZ1’s peaks appeared at 296 and 436 nm, whereas PHZ2 and PHZ3 peaked at only one position, indicating that the change in electron-giving groups affected the UV-absorption peaks; the corresponding electrochemical properties may also change, or this behavior may be related to the concentration of the oligomer solution. The latter three oligomers presented two peak locations and relatively clear heights, which may have occurred due to the prominent optical properties of benzophenone in the ultraviolet diagram, indicating that the absorption capacity of the electron-absorbing groups was more important for the optical performance of the D–A oligomers [29]. We observed that the electron-cloud distribution of the oligomer was related to the maximum ultraviolet-absorption wavelength value, and the higher the electron absorption capacity, the greater the absorption wavelength.

### 3.5. Electrochromic Properties

**Figure 4:**
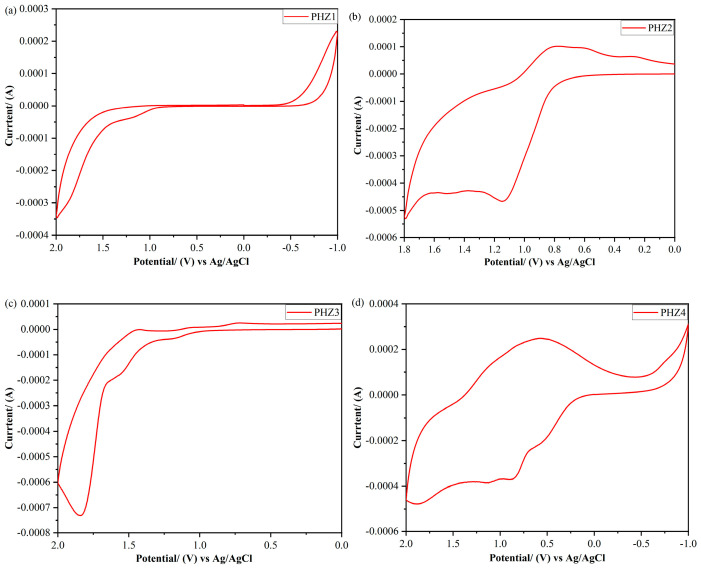

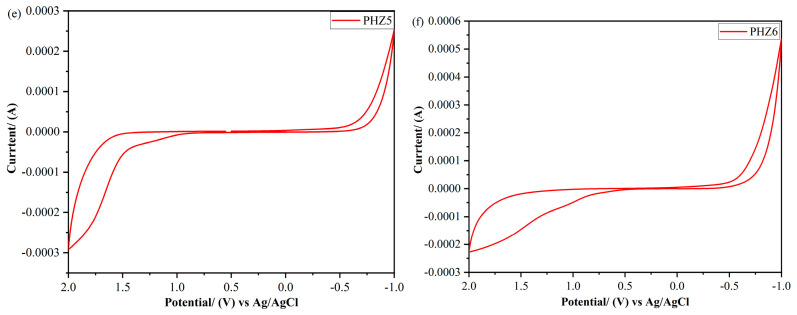
Cycle voltammetries of oligomers at 0.2 mol·L^−1^ TBAP/CH_3_CN at a scanning rate of 50 Mv · s^−1^: (**a**) PHZ1, (**b**) PHZ2, (**c**) PHZ3, (**d**) PHZ4, (**e**) PHZ5, (**f**) PHZ6.

Cyclic voltammetry (CV) was employed in this study as a reliable test for electrochromic characteristics. To create a standard three-electrode system, ITO with an oligomer film was used as the working electrode, a platinum wire as the counter electrode, and Ag/AgCl, KCl (sat) as the reference electrode. With the addition of 0.2 mol·L^−1^ of TBAP, CH_3_CN was the solvent we used [30]. Figure 4 displays all of the oligomers’ cyclic voltammetries. The figures clarify that all the oligomers have reversible electrochromic characteristics. It can also be observed that the peak value of each oligomer is different. We proved that the benzothiadiazole group and fluorene polymerization effect improve [31,32], the benzophenone and cyclopententhiophene group combination can produce a good chemical reaction, and the oligomer with alkyl side-chain modification presents better electrochemical properties. In addition, the area in the CV curve is also related to the photoelectric properties of the oligomers. The larger the area, the easier it is to be transformed into other photoelectric devices, such as materials in capacitors.

### 3.6. Quantum Chemistry Calculation

**Figure 5:**
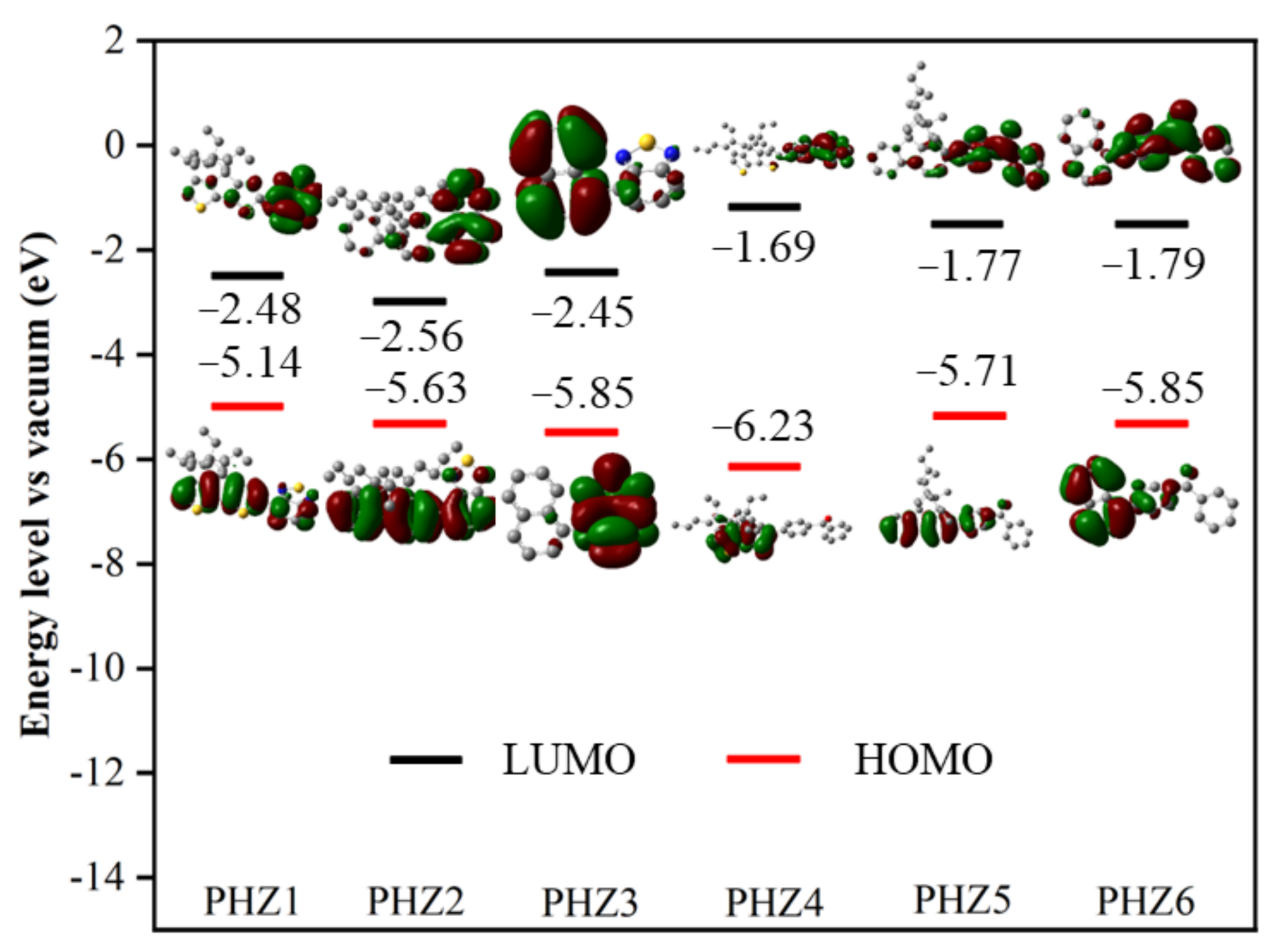
Electron-cloud densities in the leading molecular orbitals of oligomers.

The highest occupied orbitals (HOMOs) and lowest unoccupied orbitals (LUMOs) of the oligomers are important parameters to consider in the study of the properties of substances. These data allow us to analyze the mechanisms of the oxidation and reduction of oligomers. The HOMOs and LOMOs of the oligomers’ theoretical and real values are recorded in Table 4. Figure 5 shows that the electron cloud of the oligomer is mainly distributed in the electron-absorbing groups, and the band-gap values are relatively modest, which belong to the conjugated oligomer with a side-chain modification. This is because the oligomer is designed in such a way that the electron donor and electron-absorbing acceptor repeatedly alternate positions. This D–A structure can generate push and pull effects that facilitate an intramolecular charge-transfer process [33]. Therefore, the π electron is highly delocalized, and the alternations in molecular bond lengths are reduced, thus effectively controlling the band-gap value. The oligomer with this molecular structure has great advantages in the field of optoelectronics due to its adjustable photoelectric properties, various synthesis methods, high flexibility, and stable structure.

### 3.7. Spectroelectrochemical Properties

**Figure 6:**
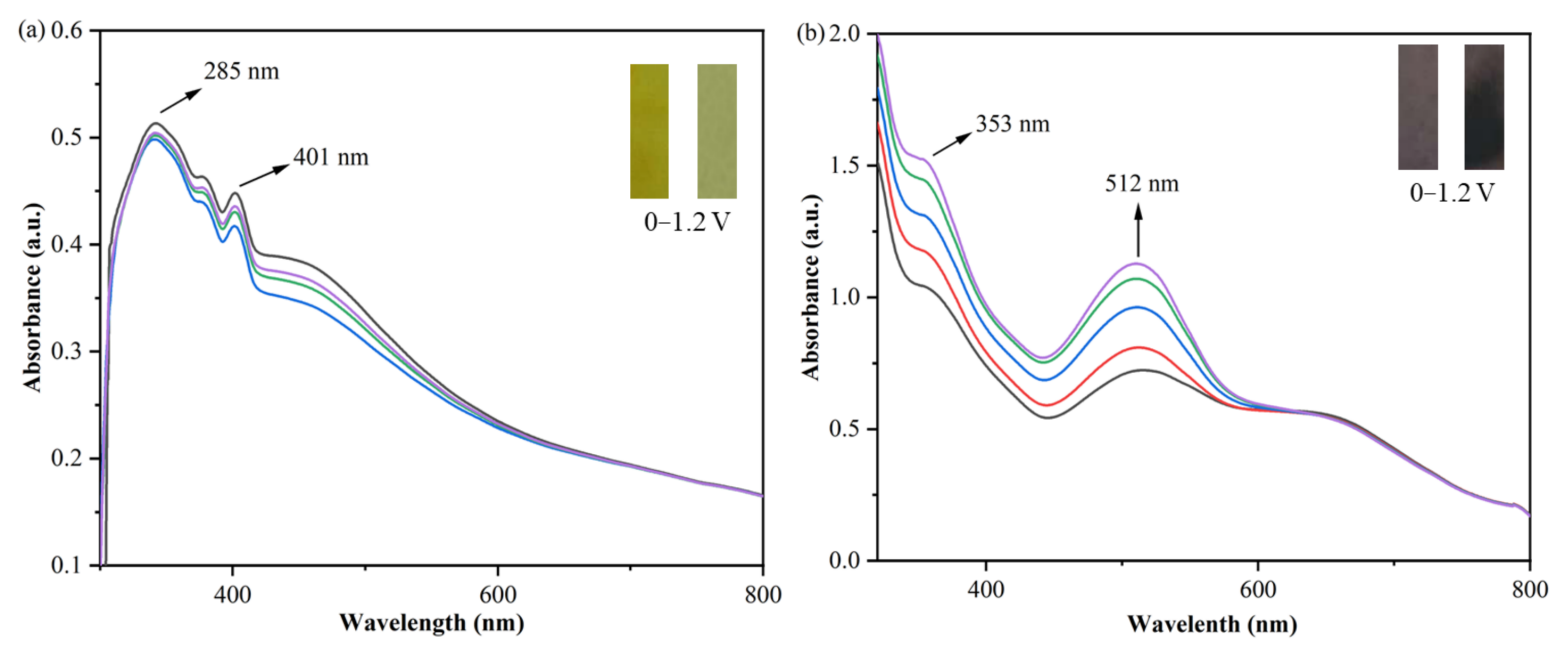

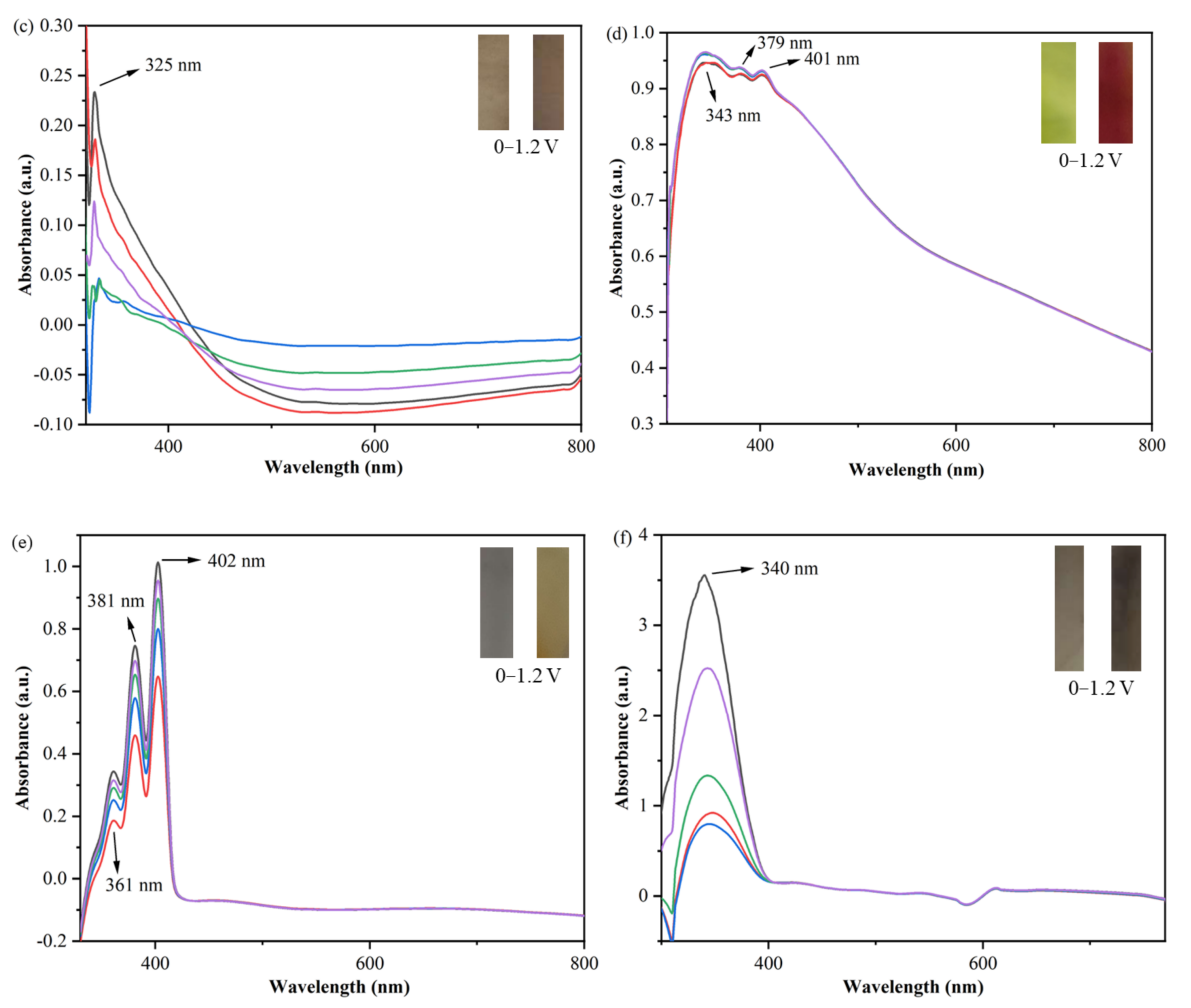
Spectroelectrochemistry results of oligomer films at different voltages in 0.2 mol·L^−1^ TBAP/CH_3_CN: (**a**) PHZ1, (**b**) PHZ2, (**c**) PHZ3, (**d**) PHZ4, (**e**) PHZ5, (**f**) PHZ6.

In this study, oligomer photovoltaic characteristics were tested under a range of voltages using UV–vis absorption spectroscopy and an electrochemical workstation. In this test, the conventional three-electrode system was still employed. As shown in Figure 6, the color changes in the six oligomers and the specific locations of the peaks are recorded in detail. Oligomers containing a cyclopententhiophene structure generally appeared yellow in color, and the color lightened under different voltages. PHZ1 and PHZ4 changed to light yellow and pink colors, respectively. This result proves that the discoloration range of the benzothiadiazole and benzophenone groups influences the color change in the entire oligomer, and the corresponding peak position in the spectroelectrochemical diagram also changes. The oligomer colors of PHZ2 and PHZ5 containing the structure of fluorene were darker prior to the REDOX reaction, and following the discoloration stage, they appeared as dark purple and earthen yellow, respectively, corresponding to the multiple peak changes evident in the figure, indicating that the oligomer can exhibit a multi-stage discoloration effect; however, it is difficult to observe this with the human eye alone. The color changes occurring in PHZ3 and PHZ6 containing naphthalene structures were similar, shifting from light to dark gray; the corresponding wavelength change was also similar, indicating that the naphthalene-containing oligomer’s color change mainly depended on the side chain of the electron-absorbing group.

### 3.8. Kinetic Study of the Oligomer Films

**Figure 7:**
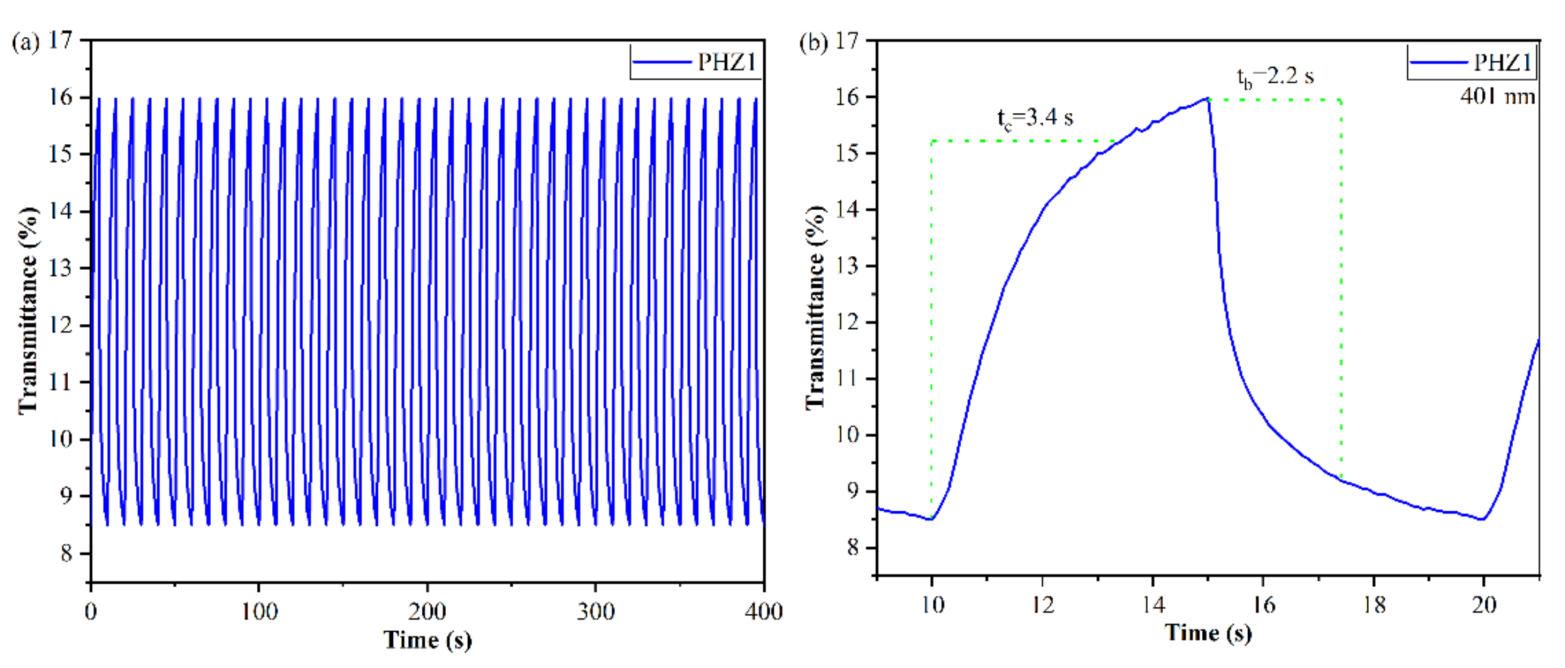

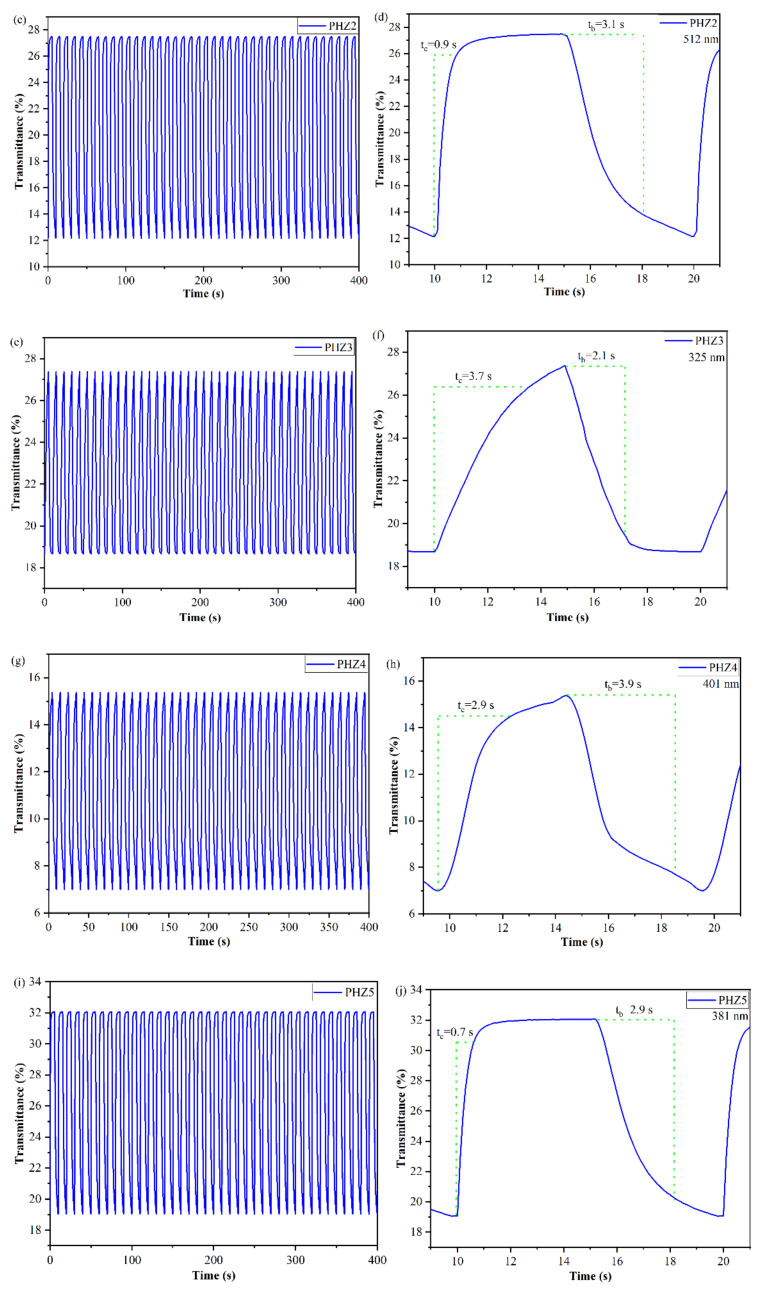

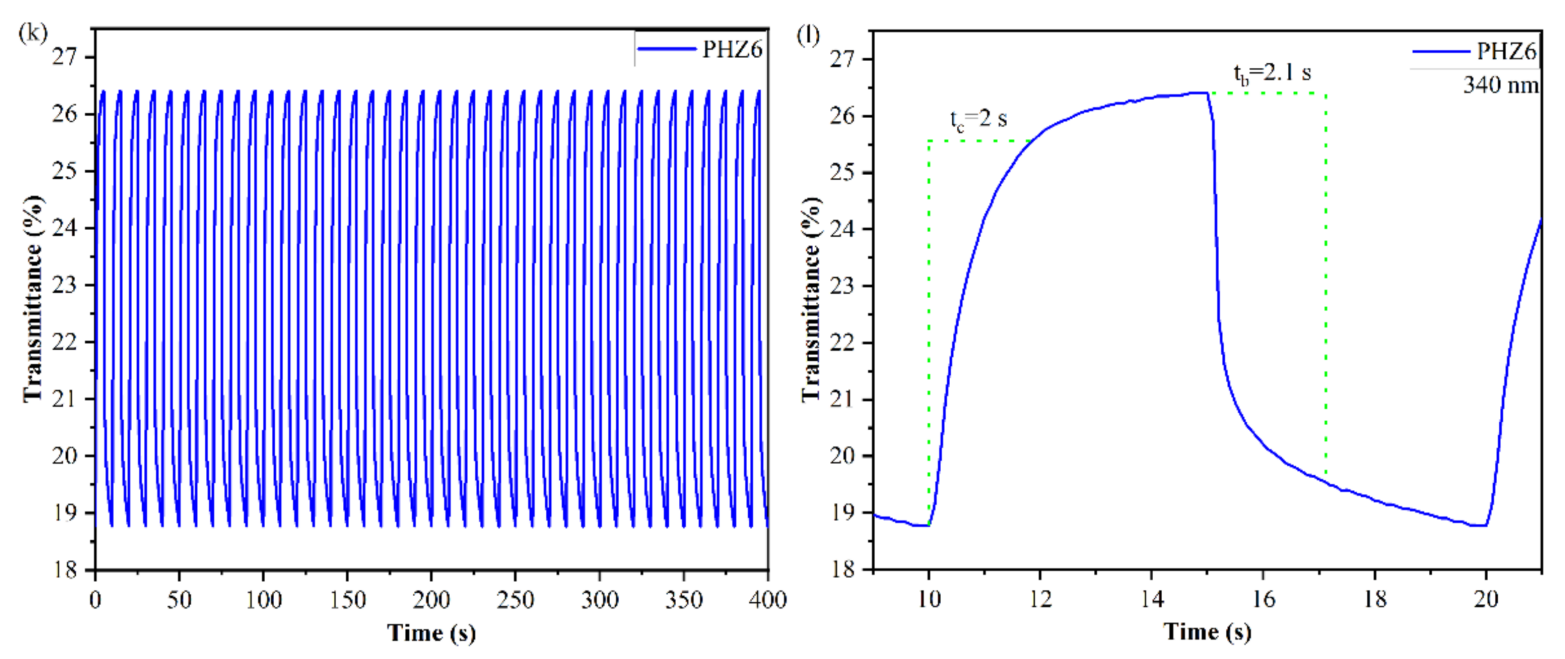
Transmissions of oligomer films: (**a**) PHZ1, (**c**) PHZ2, (**e**) PHZ3, (**g**) PHZ4, (**i**) PHZ5, (**k**) PHZ6; switching-response times: (**b**) PHZ1, (**d**) PHZ2, (**f**) PHZ3, (**h**) PHZ4, (**j**) PHZ5, (**l**) PHZ6 in 0.2 mol·L^−1^ TBAP/CH_3_CN.

The study of kinetics is a key part of electrochemical testing because it is related to the working stability of oligomers, the color-change time, and the change in transmittance activity. The electrochromic performance of the oligomer can be calculated according to the data of these parameters. Figure 7 shows the dynamic tests performed for the six oligomers for 40 stable cycles at corresponding voltages, and the corresponding switching-response times for all oligomers are also presented. The results show that PHZ2 and PHZ5 containing fluorene present relatively quick coloring times (t_c_), whereas PHZ3 and PHZ6 containing naphthalene structures have relatively short bleaching times (t_b_). All the oligomers showed good working stability behaviors and switching-response times, which proves that their electrochromic properties have good application prospects.

### 3.9. Coloration Efficiencies of Oligomers

To examine the kinetic characteristics of the ECs, the coloration efficiency (CE) was tested and is presented in this section. CE is a crucial component in successfully gauging an electrochromic material’s properties [34]. The CE value can be calculated by the following formula:

ΔOD = log (T_b_/T_c_) (1)

CE = ΔOD/Qd(2)

The corresponding electrochemical parameters of each oligomer are listed in Table 5. It can be observed that PHZ4 has the greatest color-development efficiency of 283 cm^2^·C^−1^, and the transmittance values of the two oligomers containing fluorene vary greatly. The comparison shows that the modification of the alkyl side chain has a certain effect on the electrochromic properties of the oligomer, and the improvement to the solubility is helpful for testing oligomer films.

### 3.10. Photodetector Performances of Oligomers

Bulk heterojunction oligomer photodetectors were created to test the oligomers (PHZ1, PHZ2, PHZ4) for their photodetection capabilities [35]. For photodetector devices, PC_61_BM was used as the acceptor, and three different oligomers were used as the donors. The configuration of the device was ITO/PEDOT:PSS/oligomer:PC_61_BM/Al. 

For oligomer photodetectors, the shot noise produced by the dark current can be considered to be the main source of total noise. Therefore, the specific detectivity of the photodetector can be calculated from Equation (3):

D* = R/(2qJ_d_)^1/2^ = (J_ph_/L_light_)/(2qJ_d_)^1/2^ (Jones) (3)

In the equation, J_d_ is the dark current density (A/cm^2^) and q is the absolute value of the electron charge (1.6 × 10^−19^ Coulombs), and r is the spectral responsiveness or the ratio of the photocurrent (J_ph_) to the incident-light intensity (L_light_). In our test setup, the EQE of the device can be measured directly, after which the responsiveness is calculated according to Equation (4):

R = J_ph_/L_light_ = (EQEλ)/1240(A/W) (4)

In the equation, λ (nm) is the wavelength corresponding to the EQE, therefore the specific detectivity value of the devices can be calculated by Equation (5):

D* = R/(2qJ_d_)^1/2^ = (EQEλ)/[1240(2qJ_d_)^1/2^] (Jones) (5)

The specific detectivity value calculated obtained from Equation (5) may be greater than the actual value, as the actual equipment’s noise may be louder due to the presence of other noise sources.

**Figure 8:**
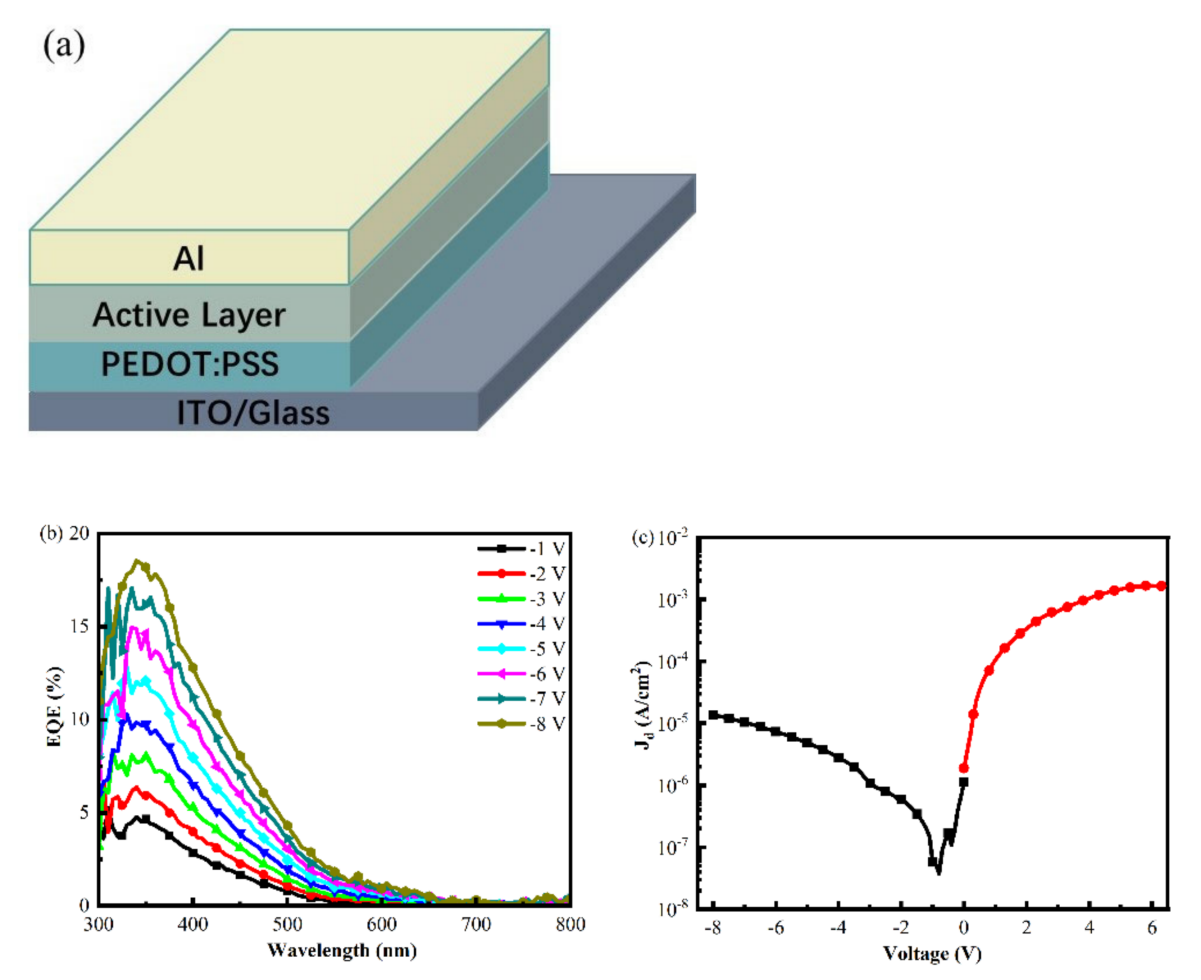

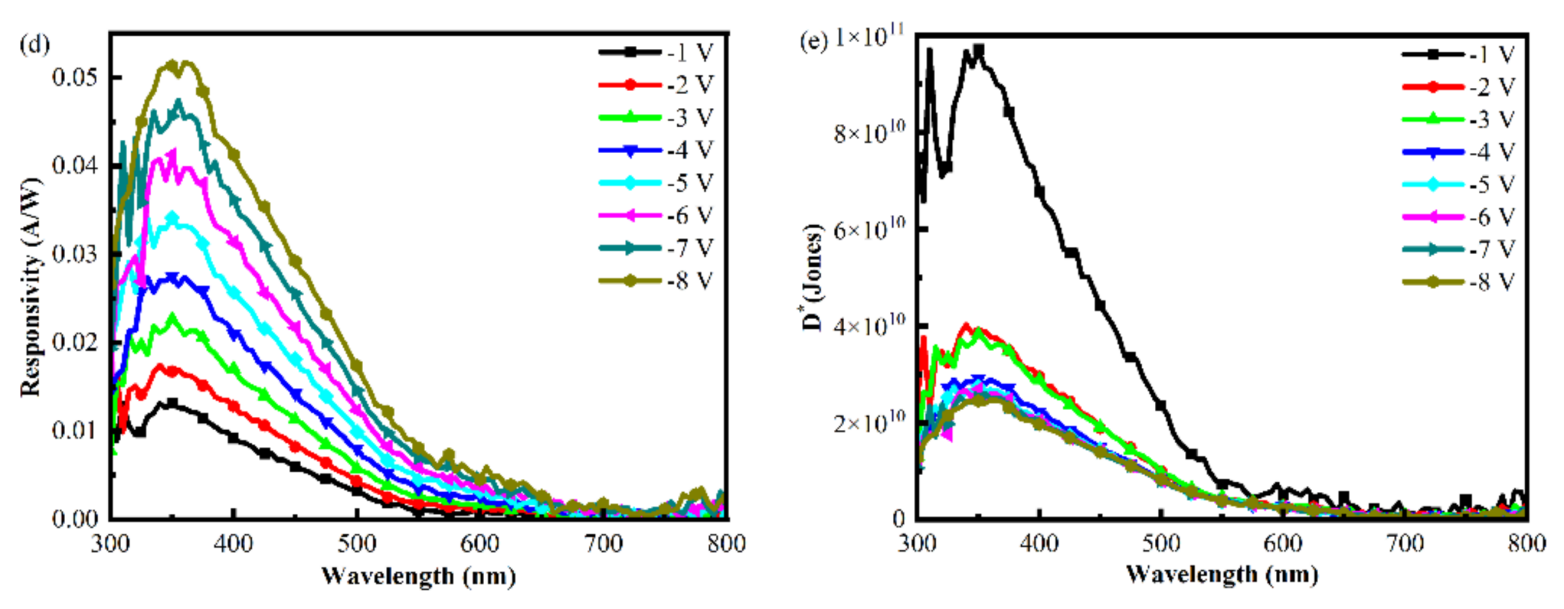
Characterizations of three oligomer-based photodetectors. Device configurations: (**a**); EQE: (**b**) PHZ4; J–V curves in dark shades: (**c**) PHZ4; responsivity values: (**d**) PHZ4; specific detectivity values: (**e**) PHZ4 (all details are presented in the Supplementary Materials).

The key properties exhibited by the photodetectors based on these three oligomers are shown in Figure 8. According to the experimental results, we can observe that the properties of the three oligomers are related to the molecular weight of the oligomeric monomer. The molecular weight of PHZ4 is the heaviest and the corresponding properties are the best. Therefore, it is inferred that the performance of the photodetector is related to the size of the doped oligomer molecules. Through our research, we discovered that photodetectors based on these three oligomers showed good gains and specific detectivities, revealing further opportunities for electrochromic materials in the photodetector industry.

## 4. Conclusions

Six conjugated oligomers containing D–A structures were successfully designed and synthesized. All the oligomers under study presented excellent electrochromic parameters and cyclic stability values, among which PHZ4 presented the best color-development efficiency (283 cm^2^·C^−1^), PHZ5 exhibited the shortest coloring time (0.7 s), and PHZ3 and PHZ6 presented the shortest bleaching times (2.1 s). In addition, D–A structures containing conducting oligomers also proved to be suitable for use in photodetectors. Based on the good specific detection rates and gains of the three kinds of photodetectors assessed in this study, the results indicate that conjugated oligomers have a good application prospect in photoelectric devices in the future.

## Figures and Tables

**Table 1:** Solubility values of oligomers in common organic solvents.

Oligomer Code	Solubility in Organic Solvents ^a^					
	DMF	DMSO	DMAC	THF	NMP	DCM
**PHZ1**	+ +	+ +	+ +	+ −	+ +	+ +
**PHZ2**	+ +	+ +	+ +	+ −	+ +	+ +
**PHZ3**	+ +	+ +	+ +	+ −	+ −	+ +
**PHZ4**	+ +	+ +	+ +	+ −	+ +	+ +
**PHZ5**	+ −	+ +	+ −	+ −	+ +	+ +
**PHZ6**	+ −	+ +	+ −	+ −	+ +	+ +

^a^ + +, soluble at room temperature; + −, slightly soluble at room temperature.

**Table 2:** Gel-permeation chromatography data for oligomers.

Oligomer Code	Mn	Mw	PDI ^a^	M ^b^	N ^c^
**PHZ1**	5067	5827	1.15	692.18	7.32
**PHZ2**	5203	6296	1.21	680.18	7.65
**PHZ3**	2153	2669	1.24	260.35	8.27
**PHZ4**	5714	7428	1.30	738.22	7.74
**PHZ5**	5468	6999	1.28	726.22	7.53
**PHZ6**	2598	3195	1.23	306.39	8.48

^a^ Oligomer dispersity index = Mw/Mn. ^b^ Oligomeric unit molecular weight. ^c^ Oligomerization degree = Mn/M.

**Table 3:** Optical properties of oligomers.

Oligomer	In Solution λ_max_ (nm)	
**PHZ1**	296	436
**PHZ2**	355	-
**PHZ3**	342	-
**PHZ4**	295	414
**PHZ5**	288	315
**PHZ6**	313	399

The data were measured in DMF at a concentration of 10^−5^ mol·L^−1^.

**Table 4:** Optical and electrochromic properties of oligomers.

**Oligomer Code**	$E_{onset}^{OX}$(V)	E_HOMO_ (eV) ^a^	E_LUMO_ (eV) ^b^	E_g_ (eV) ^c^	λ_onset_^a^ (nm) ^d^	$E_{HOMO}^{quantun}$ (eV)	$E_{LOMO}^{quantun}$ (eV)	$E_{g}^{quantun}$ (eV)
**PHZ1**	1.23	−5.66	−2.82	2.84	436	−5.14	−2.48	2.66
**PHZ2**	1.15	−5.58	−2.09	3.49	355	−5.63	−2.56	3.07
**PHZ3**	1.20	−5.63	−2.01	3.62	342	−5.85	−2.45	3.40
**PHZ4**	1.18	−5.61	−1.41	4.20	295	−6.23	−1.69	4.54
**PHZ5**	1.19	−5.62	−1.68	3.94	315	−5.71	−1.77	3.94
**PHZ6**	1.07	−5.50	−1.54	3.96	313	−5.85	−1.79	4.06

Note: E_quantum_, theorical calculation of the oligomers. ^a^ E_HOMO_ = −e (vs. Ag/AgCl + 4.43) eV. ^b^ E_LOMO_ = E_HOMO_ + E_g_. ^c^ E_g_ = 1240/λ_onset_. ^d^ Initial UV–vis absorption properties of oligomers on thin films.

**Table 5:** Electrochromic properties of oligomers.

Oligomer Code	λ_max_ ^a^ (nm)	Δ%T	t_b_^b^ (s)	t_c_^b^ (s)	ΔOD ^c^	Q_d_^d^ (mC·cm^−2^)	CE ^e^ (cm^2^·C^−1^)
**PHZ1**	401	7.4	2.2	3.4	0.27	2.1	128
**PHZ2**	512	15	3.1	0.9	0.35	1.7	206
**PHZ3**	325	8.7	2.1	3.7	0.17	1.4	121
**PHZ4**	401	8.4	3.9	2.9	0.34	1.2	283
**PHZ5**	381	13	2.9	0.7	0.23	1.5	153
**PHZ6**	340	7.6	2.1	2.0	0.15	0.9	167

^a^ Maximum-absorption wavelength of the oligomer films. ^b^ Time for 90% of the full-transmittance change. ^c^ Optical density (ΔOD) = log[T_bleached_/T_colored_]. T_bleached_ is the maximum transmittance in the neutral states, and T_colored_ is the maximum transmittance in the oxidized states. ^d^ Qd is an ejection charge tested by the experiments. ^e^ Coloration efficiency. (CE) = ΔOD/Qd.

## Data Availability

The data that support the findings of this study are available from the corresponding author upon reasonable request.

## Supplementary Materials

The following supporting information can be downloaded at: https://www.mdpi.com/article/10.3390/polym15102274/s1, Figure S1: ^1^HNMR spectrum of 4, 4-bis (2-ethylhexyl) -4h-cyclopentadithiophene; Figure S2: ^1^HNMR spectrum of M2; Figure S3: ^1^HNMR spectrum of M4; Figure S4: ^1^HNMR spectrum of PHZ1; Figure S5: ^1^HNMR spectrum of PHZ2; Figure S6: ^1^HNMR spectrum of PHZ3; Figure S7: ^1^HNMR spectrum of PHZ4; Figure S8: ^1^HNMR spectrum of PHZ5; Figure S9: ^1^HNMR spectrum of PHZ6; Figure S10: Cyclic voltammograms of Fc/Fc + reference; Figure S11: Current of oligomer films. (a) PHZ1, (b) PHZ2, (c) PHZ3, (d) PHZ4, (e) PHZ5, (f) PHZ6; Figure S12: Characterization of three oligomers-based photodetectors. EQE: (f) PHZ1, (j) PHZ2; J-V curves in dark: (g) PHZ1, (k) PHZ2; Responsivity: (h) PHZ1, (l) PHZ2; Specific detectivity: (i) PHZ1, (m) PHZ2.

## References

[1] Fu H., Zhan Y.F., Zhang L., Zhan W., Dong Y., Li W., Zhang C. (2023). Dual polymer electrochromic sunglasses with black to anti-blue-ray conversion based on new anti-blue-ray transparent polymer. Chem. Eng. J..

[2] Debnath S., Masilamani G., Agrawal A., Kumar N.R., Kumar C., Zade S.S., Bedi A. (2022). Cyclopenta[c]thiophene- and Diketopyrrolopyrrole-Based Red-Green-Blue Electrochromic Polymers. Org. Mater..

[3] Bedi A., Senanayak S.P., Das S., Narayan K.S., Zade S.S. (2012). Cyclopenta[c]thiophene oligomersbased solution processable D–A copolymers and their application as FET materials. Polym. Chem..

[4] Li F.W., Yen T.C., Liou G.S. (2021). Synthesis of high-performance electrochromic material for facile fabrication of truly black electrochromic devices. Electrochim. Acta.

[5] Vujković M.J., Etinski M., Vasić B., Kuzmanović B., Bajuk-Bogdanović D., Dominko R., Mentus S. (2021). Polyaniline as a charge storage material in an aqueous aluminum-based electrolyte: Can aluminum ions play the role of protons?. J. Power Source.

[6] Huang Q., Chen J., Shao X., Zhang L., Dong Y., Li W., Zhang C., Ma Y. (2023). New electropolymerized triphenylamine polymer films and excellent multifunctional electrochromic energy storage system materials with real-time monitoring of energy storage status. Chem. Eng. J..

[7] Schrickx H.M., Sen P., Booth R.E., Altaqui A., Burleson J., Rech J.J., Lee J.W., Biliroglu M., Gundogdu K., Kim B.J. (2021). Ultra-High Alignment of Polymer Semiconductor Blends Enabling Photodetectors with Exceptional Polarization Sensitivity. Adv. Funct. Mater..

[8] Zhang Z., Geng Y., Cao S., Chen Z., Gao H., Zhu X., Zhang X., Wu Y. (2022). Ultraviolet Photodetectors Based on Polymer Microwire Arrays toward Wearable Medical Devices. ACS Appl. Mater. Interface.

[9] Wang X., Gao S., Han J., Liu Z., Qiao W., Wang Z.Y. (2022). High-Performance All-Polymer Photodetectors Enabled by New Random Terpolymer Acceptor with Fine-Tuned Molecular Weight. ACS Appl. Mater. Interface.

[10] Lin Y.C., Chen C.H., Lin H., Li M.H., Chang B., Hsueh T.F., Tsai B.S., Yang Y., Wei K.H. (2022). Binary alloy of functionalized small-molecule acceptors with the A–DA’D–A structure for ternary-blend photovoltaics displaying high open-circuit voltages and efficiencies. J. Mater. Chem. A.

[11] Jena S.R., Choudhury J. (2022). Solar cell-coupled metallo-supramolecular polymer-based electrochromic device in renewable energy storage and on-demand usage. Sol. Energy Mater. Sol. Cells.

[12] Lin K., Wu C., Zhang G., Wu Z., Tang S., Lin Y., Li X., Jiang Y., Lin H., Wang Y. (2022). Toward High-Performance Electrochromic Conjugated Polymers: Influence of Local Chemical Environment and Side-Chain Engineering. Molecules.

[13] Jia S., Qi S., Xing Z., Li S., Wang Q., Chen Z. (2023). Effects of Different Lengths of Oligo (Ethylene Glycol) Side Chains on the Electrochromic and Photovoltaic Properties of Benzothiadiazole-Based Donor-Acceptor Conjugated Polymers. Molecules.

[14] Xu Z., Wang B., Kong L., Zhao J., Du Y. (2023). Synthesis and Characterization of Solution-Processible Donor-Acceptor Electrochromic Conjugated Copolymers Based on Quinoxalino[2’,3’:9,10]phenanthro[4,5-abc]phenazine as the Acceptor Unit. Polymers.

[15] Zhang W., Feng X., Zhang C., Jia Q., Liu X., Zhou K. (2023). Activity of N–H in phenothiazine derivatives: Synthesis and applications in fluoride ions sensing and electrochromism. J. Mater. Chem. C.

[16] Santra D.C., Nad S., Malik S. (2018). Electrochemical polymerization of triphenylamine end-capped dendron: Electrochromic and electrofluorochromic switching behaviors. Electroanal. Chem..

[17] Crocomo P.Z., Okazaki M., Hosono T., Minakata S., Takeda Y., Data P. (2022). Dibenzophenazine-Based TADF Emitters as Dual Electrochromic and Electroluminescence Materials. Chem. Eur. J..

[18] Dong D., Djaoued H., Vienneau G., Robichaud J., Brown D., Brüning R., Djaoued Y. (2020). Electrochromic and colorimetric properties of anodic NiO thin films: Uncovering electrochromic mechanism of NiO. Electrochim. Acta.

[19] Carbas B.B., Özbakır S., Kaya Y. (2023). A comprehensive overview of carbazole-EDOT based electrochromic copolymers: A new candidate for carbazole-EDOT based electrochromic copolymer. Synth. Met..

[20] Topal S., Savlug Ipek O., Sezer E., Ozturk T. (2022). Electrochromic-Hybrid energy storage material consisting of triphenylamine and dithienothiophene. Chem. Eng. J..

[21] Wang J.L., Sheng S.Z., He Z., Wang R., Pan Z., Zhao H.Y., Liu J.W., Yu S.H. (2021). Self-Powered Flexible Electrochromic Smart Window. Nano Lett..

[22] Yu X., Chang M., Chen W., Liang D., Lu X., Zhou G. (2020). Colorless-to-Black Electrochromism from Binary Electrochromes toward Multifunctional Displays. ACS Appl. Mater. Interfaces.

[23] Zhang W., Li H., Elezzabi A.Y. (2021). Electrochromic Displays Having Two-Dimensional CIE Color Space Tunability. Adv. Funct. Mater..

[24] Gao Z., Kong L., Ming S., Du H., Zhang Y., Zhao J. (2023). D-A type ambipolar electrochromic copolymers based on dithienopyrrole, 3,4-propylenedioxythiophene and benzotriazole units with dual fading processes. Eur. Polym. J..

[25] Hsiao S.H., Chen Y.Z. (2017). Electroactive and ambipolar electrochromic polyimides from arylene diimides with triphenylamine N -substituents. Dye. Pigment..

[26] Sk B., Sarkar M., Singh K., Sengupta A., Patra A. (2021). UV to NIR multistate electrochromism and electrofluorochromism in dibenzophenazine-arylamine derivatives. Chem. Commun..

[27] Schmatz B., Ponder J.F., Reynolds J.R. (2017). Multifunctional triphenylamine polymers synthesized via direct (hetero) arylation polymerization. J. Polym. Sci. Pol. Chem..

[28] Zhang H.Y., Zhao X.F., Bai J., Hou Y.J., Wang S.H., Wang C., Ma D.G. (2019). Ternary Memory Devices Based on Bipolar Copolymers with Naphthalene Benzimidazole Acceptors and Fluorene/Carbazole Donors. Macromolecules.

[29] Çakal D., Ertan S., Cihaner A., Önal A.M. (2018). Synthesis and electrochemical polymerization of D-A-D type monomers with thieno 3,4-c] pyrrole-4,6-dione acceptor unit. Dye. Pigment..

[30] Bayat M., Izadan H., Santiago S., Estrany F., Dinari M., Semnani D., Alemán C., Guirado G. (2021). Study on the electrochromic properties of polypyrrole layers doped with different dye molecules. Electroanal. Chem..

[31] Mei J., Heston N.C., Vasilyeva S.V., Reynolds J.R. (2009). A Facile Approach to Defect-Free Vinylene-Linked Benzothiadiazole-Thiophene Low-Bandgap Conjugated Polymers for Organic Electronics. Macromolecules.

[32] Beaujuge P.M., Vasilyeva S.V., Ellinger S., McCarley T.D., Reynolds J.R. (2009). Unsaturated Linkages in Dioxythiophene−Benzothiadiazole Donor−Acceptor Electrochromic Polymers: The Key Role of Conformational Freedom. Macromolecules.

[33] Fu W.A., Chen H.J., Yi X.G., Zhang R., Liu J. (2022). Electrochemical polymerization of D-A-D type monomers consisting of triphenylamine and benzo[1,2-b:4,5-b′]dipyrazine units for multicolor electrochromism. Eur. Polym. J..

[34] Zohrevand N., Madrakian T., Ghoorchian A., Afkhami A. (2022). Simple electrochromic sensor for the determination of amines based on the proton sensitivity of polyaniline film. Electrochim. Acta.

[35] Feng S.Y., Liu Z.T., Feng L.Z., Wang G.C., Liu B.D., Zhang X.L. (2023). High-performance self-powered ultraviolet photodetector based on Ga_2_O_3_/GaN heterostructure for optical imaging. J. Alloys Compd..

