# Supplementary material for: D–A Structural Oligomers Containing Benzothiadiazole or Benzophenone as Novel Multifunctional Materials for Electrochromic and Photodetector Devices
Source: Polymers (Basel). 2023 May 11;15(10):2274. doi: 10.3390/polym15102274 (PMC10221385; doi:10.3390/polym15102274)
Supplement: Supplementary file 1 [file polymers-15-02274-s001.zip › polymers-2348196-supplementary.pdf]

Supplementary data

D–A Structural Oligomers Containing Benzothiadiazole or Benzophenone as Novel Multifunctional Materials for Electrochromic and Photodetector Devices

Zipeng He [1], Binhua Mei [1], Hongmei Chu [1], Yanjun Hou [1,*] and Haijun Niu [2,*]

1. Key Laboratory of Chemical Engineering Process and Technology for High-Efficiency Conversion, College of Heilongjiang Province, Heilongjiang University, Harbin 150080, China
2. Key Laboratory of Functional Inorganic Material Chemistry, Ministry of Education of the People's Republic of China, Heilongjiang University, Harbin 150080, China

* Correspondence: houyj@hlju.edu.cn (Y.H.); Correspondence: haijunniu@hotmail.com (H.N.)

^{1}H NMR and ^{13}C NMR

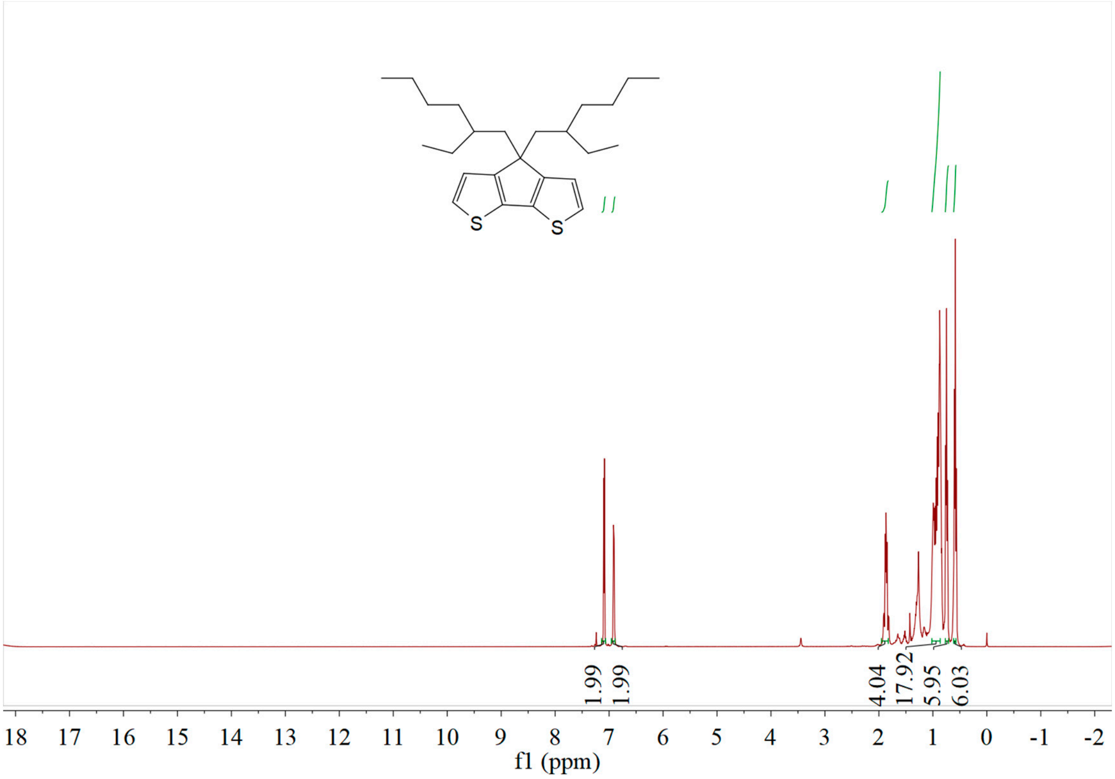

Figure S1. ^{1}HNMR spectrum of 4, 4-bis (2-ethylhexyl) -4h-cyclopentadithiophene.

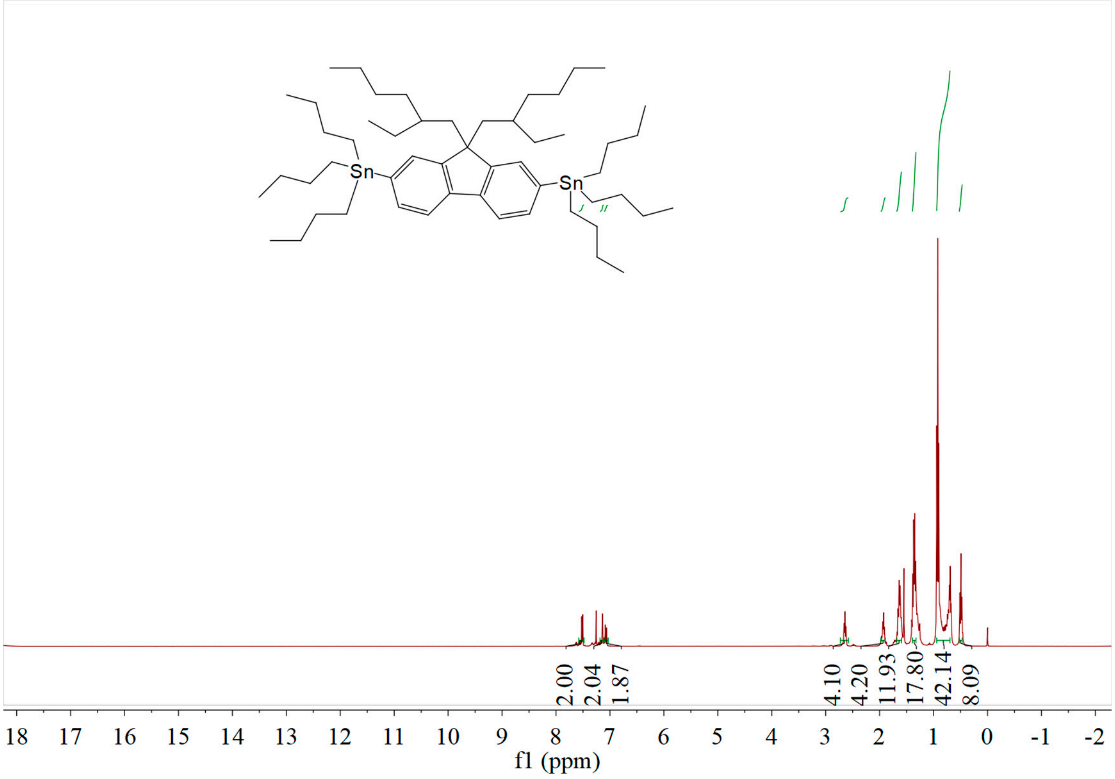

Figure S2. ^{1}HNMR spectrum of M2.

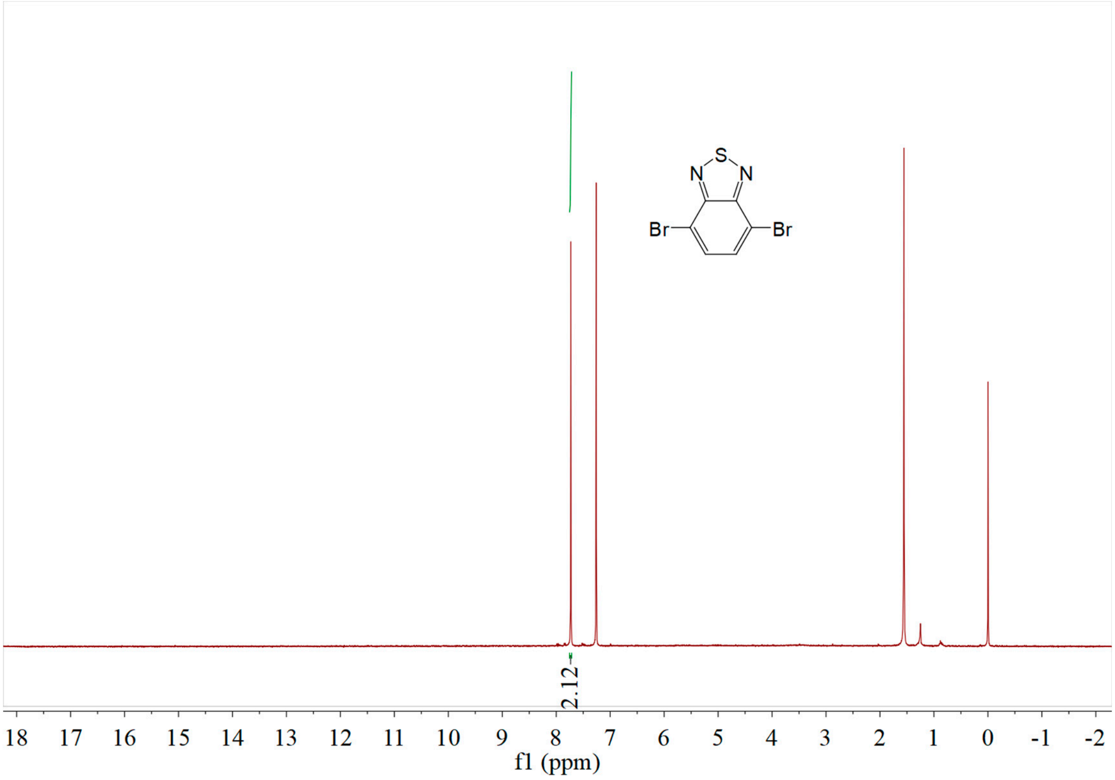

Figure S3. ^{1}HNMR spectrum of M4.

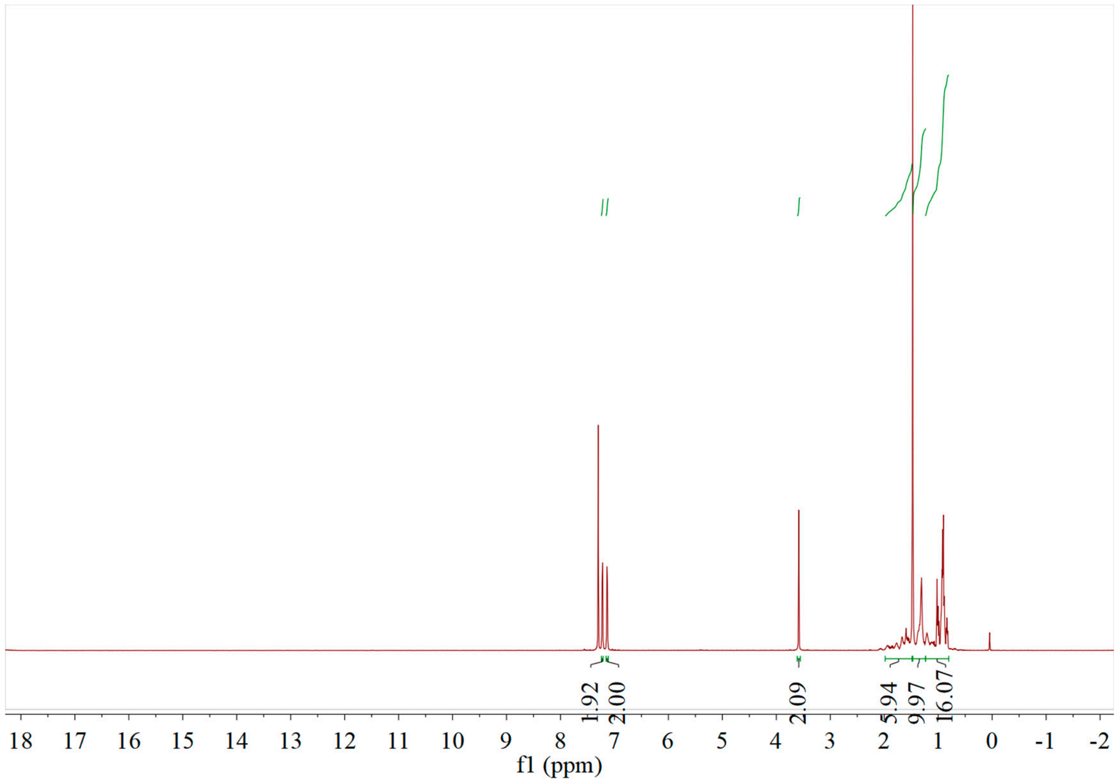

Figure S4. ^{1}HNMR spectrum of PHZ1.

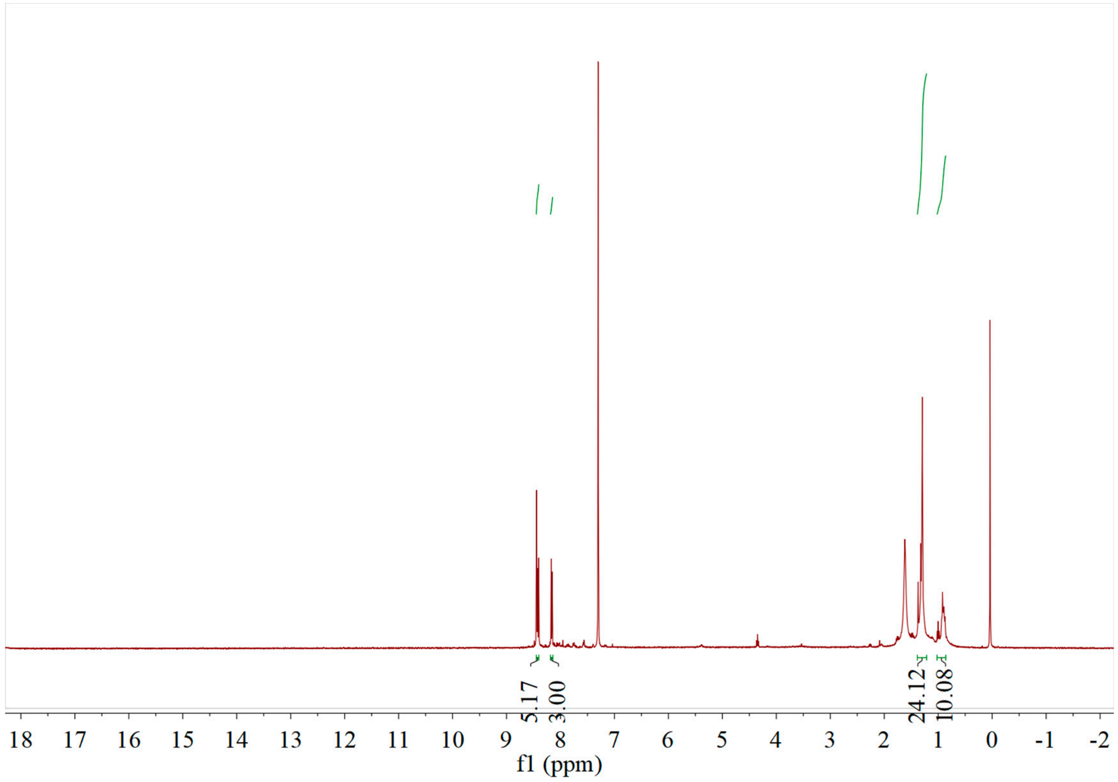

Figure S5. ^{1}HNMR spectrum of PHZ2.

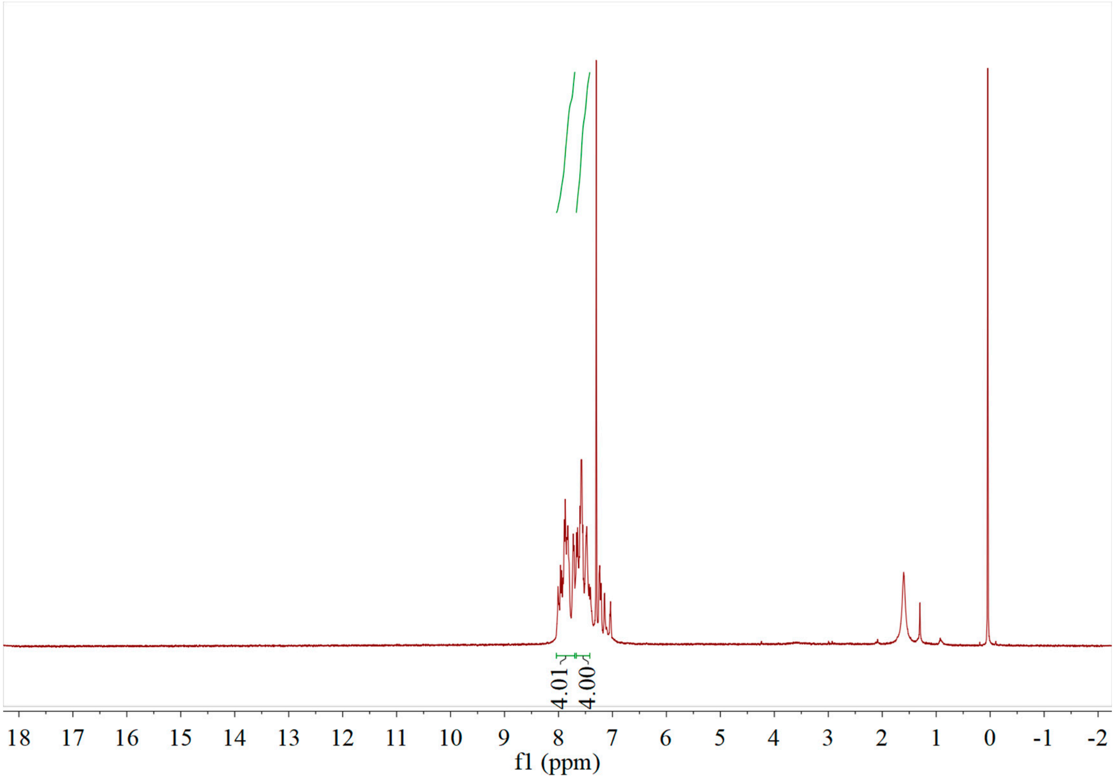

Figure S6. ^{1}HNMR spectrum of PHZ3.

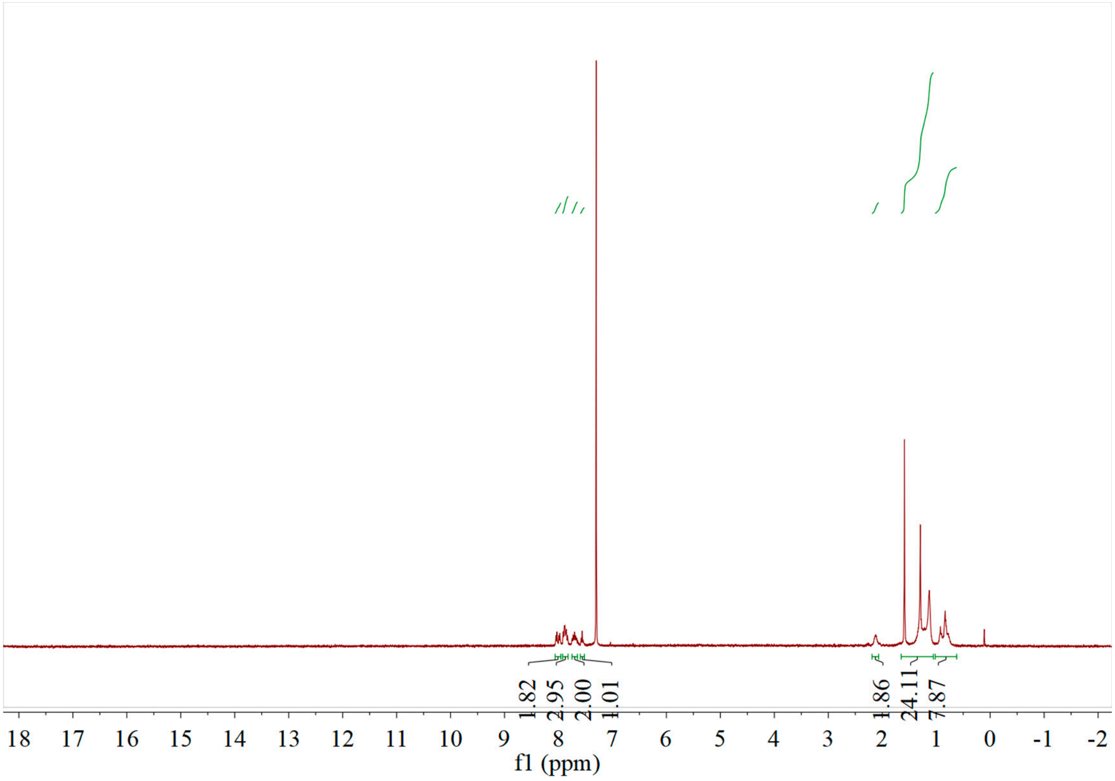

Figure S7. ^{1}HNMR spectrum of PHZ4.

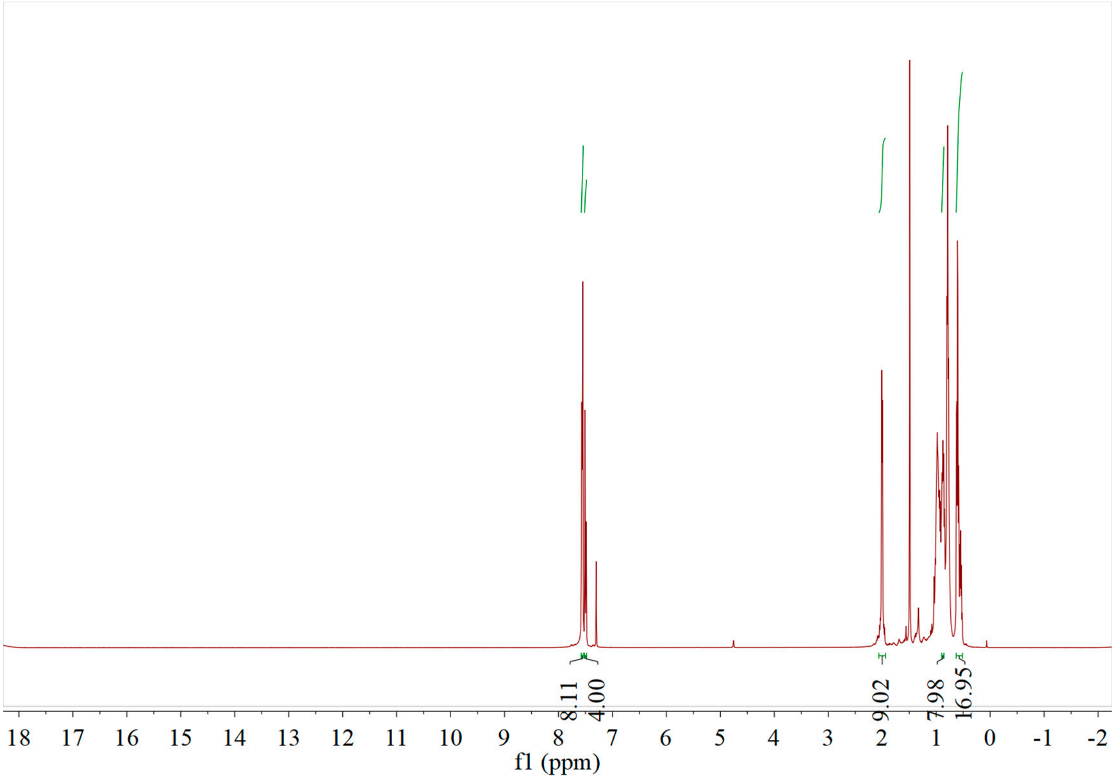

Figure S8. ^{1}HNMR spectrum of PHZ5.

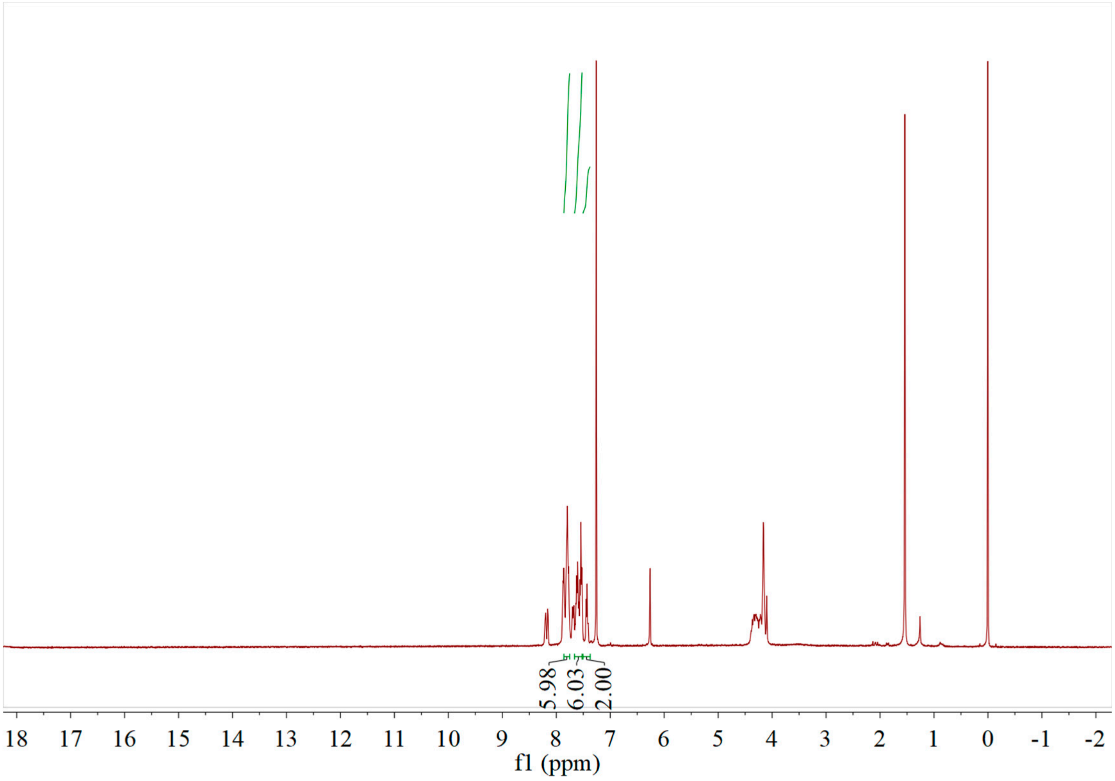

Figure S9. ^{1}HNMR spectrum of PHZ6.

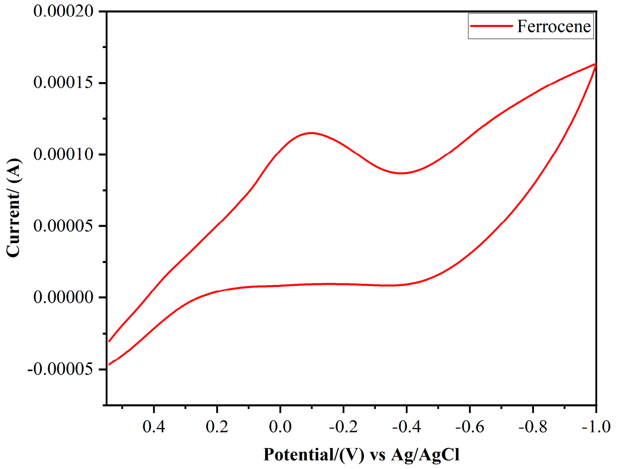

Figure S10. Cyclic voltammograms of Fc/Fc + reference.

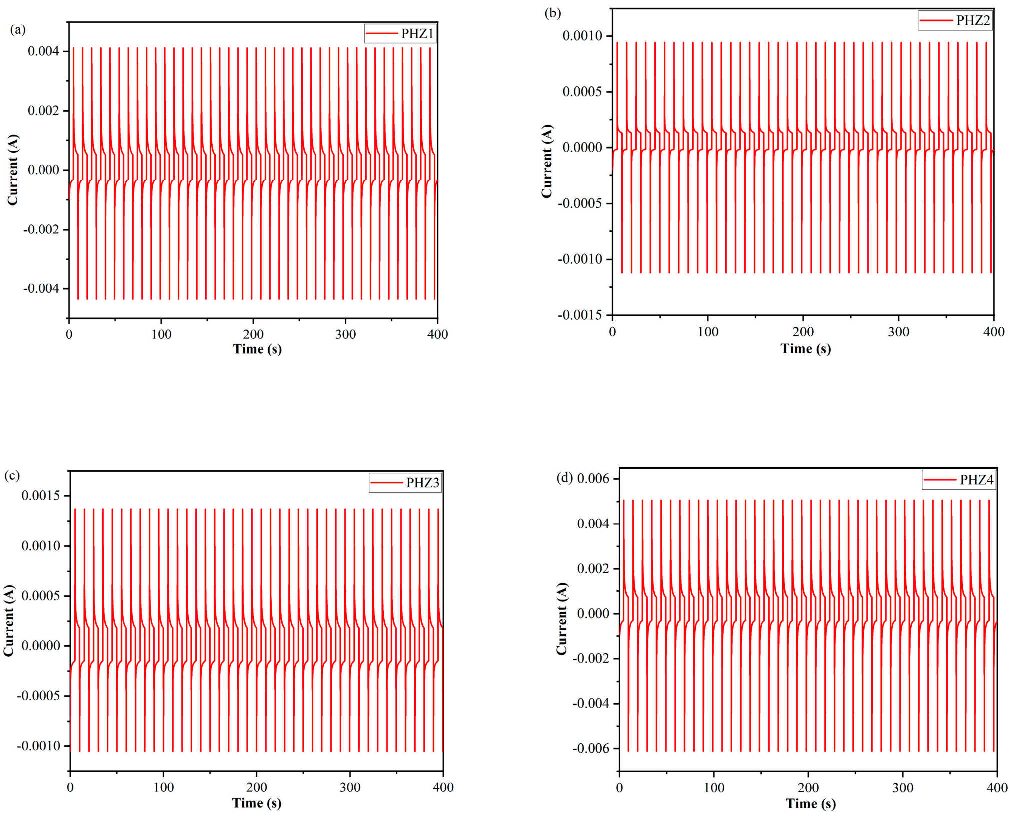

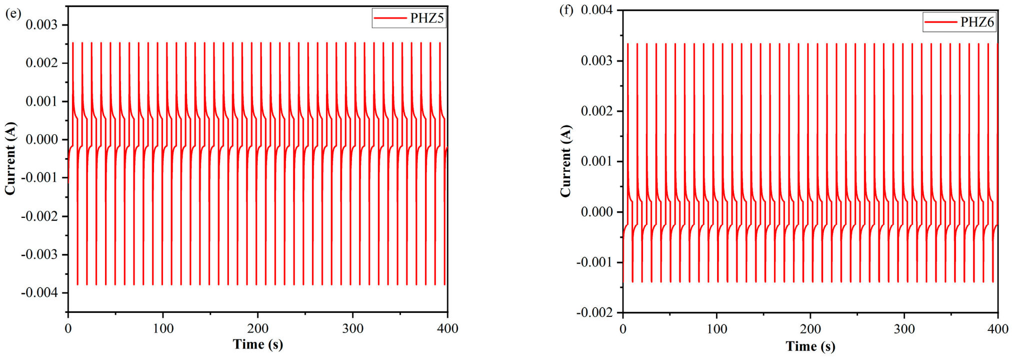

Figure S11. Current of oligomer films. (a) PHZ1, (b) PHZ2, (c) PHZ3, (d) PHZ4, (e) PHZ5, (f) PHZ6.

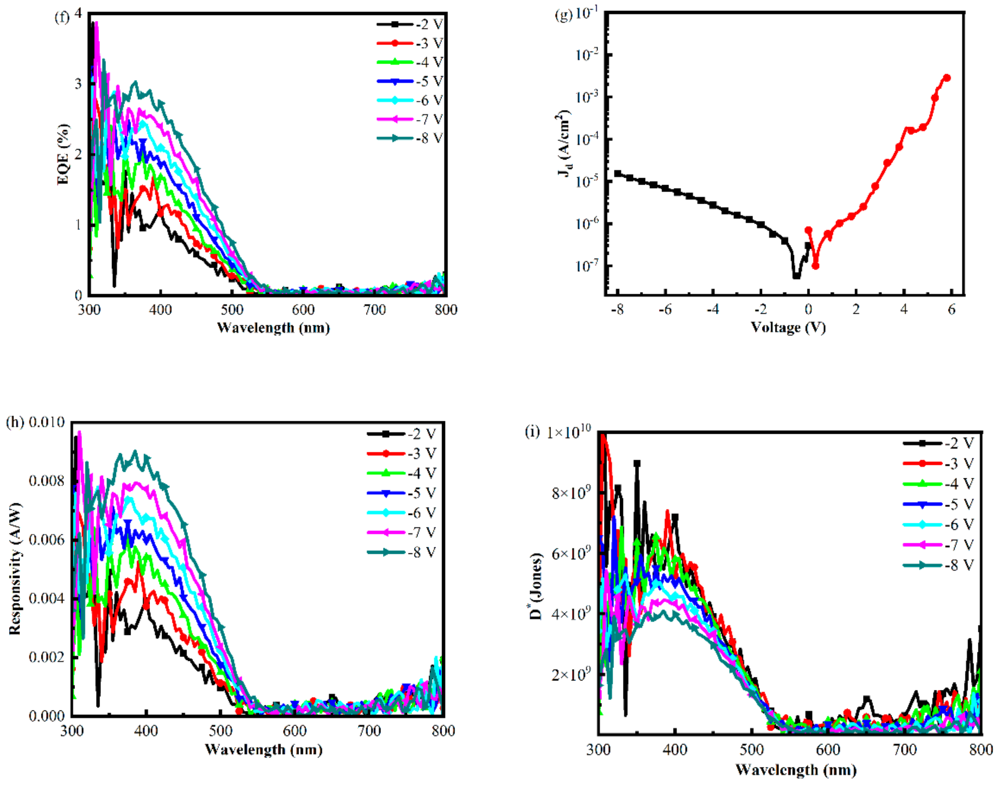

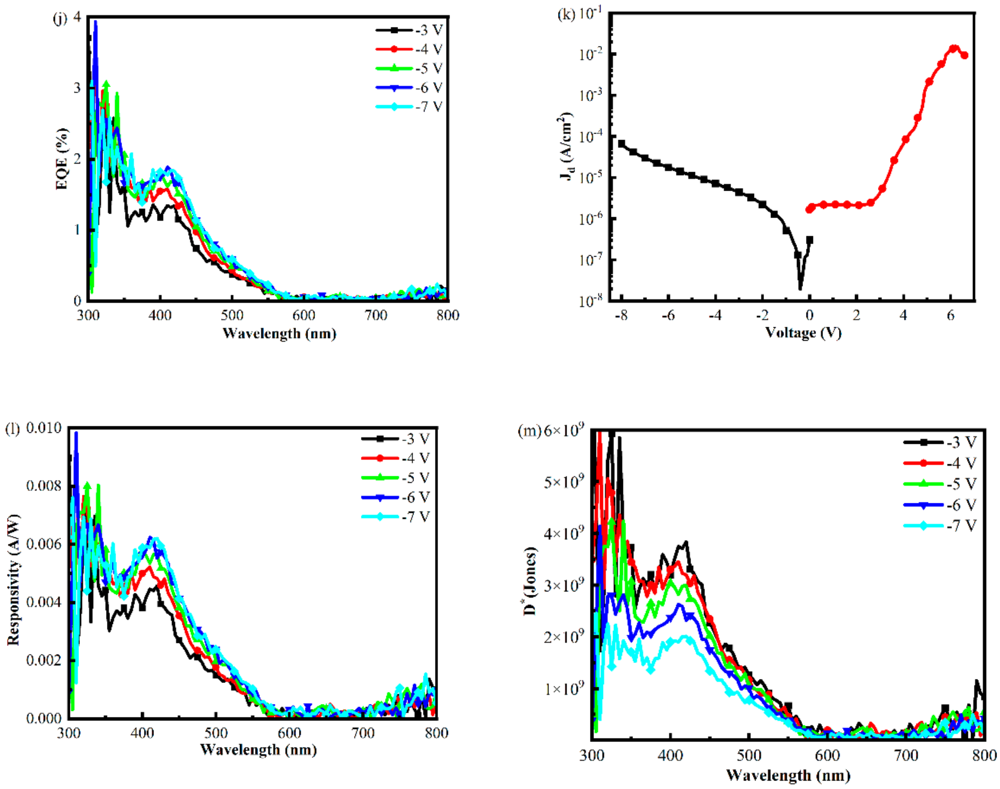

Figure S12. Characterization of three oligomers-based photodetectors. EQE: (f) PHZ1, (j) PHZ2; J-V curves in dark: (g) PHZ1, (k) PHZ2; Responsivity: (h) PHZ1, (l) PHZ2; Specific detectivity: (i) PHZ1, (m) PHZ2.

Preparation of oligomer films, photodetectors devices fabrication and characterization

Preparation of oligomer films: ITO conductive glasses needed to be treated before making the films. Acetone, ethanol, and deionized water were used to ultrasonically clean the ITO conductive glasses, respectively. 5 mL of NMP was used to dissolve 10 mg of the oligomers. At 900 rpm for 20 s, the solution was spin-coated onto the ITO glass surface. On the ITO glass surface, the solution was spin coated for 20 s at 900 rpm, and then for 60 s at 3000 rpm, a uniform coating was achieved. The solvent was then removed in a vacuum oven to produce the film. The identical method was used to

prepare all devices.

Device fabrication. The mixture of PHZ4 and $PC_{61}BM$, which has a weight ratio of 1:2, was mixed in a solution of chlorobenzene at a concentration of 10 mg/mL, heated to 60°C, and stirred for 3 hours as an illustration of a photodetector based on PHZ4. The active layer was around 120 nm thick. The ITO glass was treated with a plasma cleaner for 4 minutes after being sonicated with the HELLMA lotion for 100 minutes, rinsed with deionized water, dried in a drying oven at 120°C for 1 hour, and then rinsed with deionized water. The PEDOT:PSS solution was spin-coated onto the ITO surface at 4000 rpm for 40 s to create a 30 nm layer, which was then dried at 110°C for 30 minutes to create photodetectors with the structure ITO/PEDOT:PSS/PHZ4:$PC_{61}BM$/Al. In the nitrogen-protected glovebox, the active layer was spin-coated onto the PEDOT:PSS film for 60 s at 1000 rpm. Finally, thermal evaporation under vacuum at a pressure of 1.5×10^{-4} Pa was used to deposit the Al thin layer. Similar to those above, other photodetectors were also created.

Device characterization. To avoid electrostatic interference during all measurements in the air under ambient circumstances, the unencapsulated devices were housed in a metal enclosure. The Beijing 7-Star Optical Instruments Co. equipment was used to measure EQE. Prior to being irradiated on the device, the incident light from the 250 W halogen tungsten lamp was first chopped at 30 Hz and then separated into monochromatic light beams with an average power in the microwatt range. A calibrated silicon photodetector and an InGaAs photodetector from Humamatsu were utilized as references prior to each experiment. Data on dark current density and voltage were

collected using a Keithley 236 Source Measure instrument.

The HOMOs and LOMOs of the polymers' real values calculation

Table 4 showed the initial absorption(λs) and initial oxidation potential of oligomer films. We chose ferrocene and ferrocene ions (FC/FC+) as reference electrodes. The international standard was that the redox potential of FC/FC^{+} in vacuum is 4.80 eV. To obtain a more accurate redox potential, the cyclic voltammetry of FC/FC+ was measured (Figure S10). The energy levels of HOMO and LUMO were obtained by the following equations:

In the first step, the HOMO energy level was obtained through the initial oxidation voltage.

$$E_{HOMO} = -e\ (vs\ Ag/AgCl + 4.43)\ eV \qquad (1)$$

In the second step, the λ_{onset} of the oligomer film was known to obtain E_g:

$$E_g = 1240/\lambda_{onset} \qquad (2)$$

In the final step, the data obtained above are brought into the following equation:

$$E_{LUMO} = E_{HOMO} + E_g \qquad (3)$$
